# Comprehensive Analysis Identified Mutation-Gene Signature Impacts the Prognosis Through Immune Function in Hepatocellular Carcinoma

**DOI:** 10.3389/fonc.2022.748557

**Published:** 2022-03-04

**Authors:** Zhuo Lin, Qian Xu, Xian Song, Yuan Zeng, Liuwei Zeng, Luying Zhao, Jun Xu, Dan Miao, Zhuoyan Chen, Fujun Yu

**Affiliations:** ^1^Laboratory Animal Centre, The First Affiliated Hospital of Wenzhou Medical University, Wenzhou, China; ^2^Department of Gastroenterology, The First Affiliated Hospital of Wenzhou Medical University, Wenzhou, China

**Keywords:** hepatocellular carcinoma, drug resistance, mutation gene, overall survival, immune status, cell cycle pathway

## Abstract

**Background:**

Hepatocellular carcinoma (HCC) is a life-threatening and refractory malignancy with poor outcome. Genetic mutations are the hallmark of cancer. Thus far, there is no comprehensive prognostic model constructed by mutation-gene transcriptome in HCC. The prognostic value of mutation-gene signature in HCC remains elusive.

**Methods:**

RNA expression profiles and the corresponding clinical information were recruited from The Cancer Genome Atlas (TCGA) and International Cancer Genome Consortium (ICGC) databases. The least absolute shrinkage and selection operator (LASSO) Cox regression analysis was employed to establish gene signature. Kaplan–Meier curve and time-dependent receiver operating characteristic curve were implemented to evaluate the prognostic value. The Wilcoxon test was performed to analyze the expression of immune checkpoint genes, cell cycle genes, and tumor drug resistance genes in different risk groups. Finally, quantitative real-time PCR (qRT-RCR) and immunohistochemistry (IHC) were performed to validate the mRNA and protein expression between HCC and adjacent nontumorous tissues in an independent cohort.

**Results:**

A prognostic model consisting of five mutated genes was established by LASSO Cox regression analysis. The prognostic model classified patients into high- and low-risk groups. Compared with the low‐risk group, patients in the high‐risk group had significantly worse survival results. The prognostic model can accurately predict the overall survival of HCC patients and predict overall survival more accurately when combined with stage. Furthermore, the immune checkpoint genes, cell cycle genes, and tumor drug resistance genes were higher expressed in the high-risk group compared in the low-risk group. In addition, the expression level of prognostic signature genes was validated in an independent sample cohort, which was consistent with RNA sequencing expression in the TCGA database.

**Conclusion:**

The prediction model of HCC constructed using mutation-related genes is of great significance for clinical decision making and the personalized treatment of patients with HCC.

## Introduction

Liver cancer ranks sixth among the most common type of malignant tumor and is the second most common cause of tumor-related mortality worldwide (1). Despite advances being made in surgery, radiotherapy, chemotherapy, and other potentially curative treatment of hepatocellular carcinoma (HCC), it remains a formidable threat to human health (2). Most patients are diagnosed when the metastatic process is already present. The frequency of tumor recurrence, metastasis, and drug resistance led to the unsatisfactory 5-year survival rate of HCC patients (3). Conventional models based on vascular invasion, tumor-node-metastasis staging, and other parameters can help predict HCC prognosis (4). However, clinical heterogeneity caused by the simultaneous presence of two life-threatening diseases, cancer, cirrhosis, etc. often affects the effect of routine prognosis assessment.

Genetic mutations are the hallmark of cancer, resulting in a change in cell fate. The process of cell mutation reflects what happens in the cell, which helps us to understand the biological process of these mutations. In general, tumor occurrence is thought to require two to eight so-called driver gene mutations, as well as numerous passenger gene mutations (5, 6). Liver cancer is associated with a high degree of genomic instability and mutations of multiple genes. Metastasis and recurrence of HCC is a multistep and multifactorial process, including HCC oncogene activation, tumor suppressor gene inactivation, and mismatch repair gene mutation (7–10). Almost 30% of HCC, for example, harbor p53 mutation, which is associated with increased invasiveness, recurrence, and decreased survival rate (11, 12). Mutations in the TERT promoter, ARID1A, CDKN2A, CTNNB1, AXIN1, and CCND1 are very common in HCC (13–15). However, despite the importance of these gene mutations, they are rarely applied in routine clinical practice due to high cost and limited availability.

Next-generation sequencing technology has been widely exploited in biological studies. Genome-wide approaches provide detailed information of disease diversity, bringing a new dimension to disease diagnosis and prognosis evaluation. The Cancer Genome Atlas (TCGA) program provides whole-genome sequencing data and clinical data for 33 types of cancer (16). HCC is known to be a heterogeneous disease with multifactorial etiology, diverse characteristics, and clinical outcomes. Thus, it is necessary to establish a reliable prognostic model to monitor HCC patients and subsequently optimize the clinical treatment decision. It is well known that alpha-fetoprotein (AFP) was the clinically important tumor marker for HCC diagnosis and prognosis (17, 18). Meanwhile, some valuable biomarkers in HCC prognosis prediction were identified based on high-throughput data (19, 20). It has been reported that ferroptosis-related gene signature (21) and immune-related gene signature (22) constructed by transcriptome can predict the overall survival of HCC patients, but they do not provide a multifaceted dissection of the mechanisms. Thus far, a comprehensive prognostic evaluation model constructed by mutation-gene transcriptome has not been established to evaluate the prognosis, immune response, and tumor vascular growth of HCC patients. Therefore, the prognostic value and related mechanism of mutation-gene signature have not been explored. Identification and comprehensive analysis of mutation-gene signature for prognosis is meaningful in HCC.

In the current work, a cluster of genes with frequent mutations in HCC was screened from TCGA and International Cancer Genome Consortium (ICGC) databases. We then constructed a mutation-gene signature associated with overall survival (OS) in TCGA database. The mutation-gene signature has a capability to discriminate the immune status and tumor drug resistance in two subgroups. When integrated with the TNM stages, the prognostic performance of the gene signature is superior to that of a single biomarker. In addition, the prognostic signature generated from this study had robust validity in both the discovery cohort and the validation cohort. Moreover, the expression levels of the prognostic genes were validated in an independent sample cohort. The gene signature constructed in this study is conducive to cover the imperfection of the current prognosis evaluation of HCC.

## Materials and Methods

### Data Retrieval and Processing

The RNA sequence data and corresponding clinical information of 370 HCC patients were manually downloaded from TCGA data portal (https://portal.gdc.cancer.gov/). The samples of 231 HCC patients were obtained from the International Cancer Genome Consortium (ICGC) database (https://dcc.icgc.org/projects/LIRI-JP) for further validation. The 500 top mutation genes of HCC retrieved from TCGA and the ICGC databases are presented in **Supplementary Table 1**.

### Gene-Signature Development

The different expression of mutation genes between HCC and adjacent normal tissues was analyzed using the “limma” R package, with a false-discovery rate (FDR) of <0.05. Univariate Cox regression analysis was performed on the different expression genes (DEGs) to obtain DEGs significantly related to survival. Mutation-related genes then associated with HCC patient survival were analyzed by least absolute shrinkage and selection operator (LASSO) Cox regression method to remove confounding factors and reduce the number of genes in the risk model. A more refined Cox model was initially generated by employing the penalized maximum likelihood method, which can compress some coefficients and set others strictly equal to 0. The formula was established as follows: risk score = e^sum (each gene’s expression × corresponding coefficient)^. Based on the median risk score, the patients in TCGA dataset were separated into high- and low-risk groups. The patients in the ICGC dataset were divided into two subgroups according to the median risk score of TCGA. PCA and t-distributed stochastic neighbor embedding (t-SNE) analysis were performed with “Rtsne” and “ggplot2” R packages to explore the distribution of two subgroups. The Kaplan–Meier method was used to evaluate the relevance of overall survival in risk score groups. The time-dependent ROC curves were developed to evaluate the predictive value of the prognostic model at 1, 2, and 3 years. Furthermore, the value of independently prognostic for risk score was investigated by univariate and multivariate Cox regression analysis.

### Immune Status Analysis

The single-sample gene set enrichment analysis (ssGSEA) program was applied to evaluate the relationship between immune cell infiltration, immune pathway activity, immune function, and immune-related risk characteristics, so as to establish immune-related term enrichment scores. The scores corresponding to immune-related terms were determined for HCC patients. The Wilcoxon test was used to analyze the scores between high- and low-risk groups. In addition, the association of the risk score with immune checkpoint gene expression was also analyzed by the Wilcoxon test. The immune exclusion ability of HCC was analyzed through the TIDE database (://tide.dfci.harvard.edu/login). The model was subsequently applied to IMvigor210 trial, a phase II trial used to assess the activity of the PD-L1 antibody in patients with metastatic UC (23). The immune exclusion levels of the two subgroups and immune response of the two groups were analyzed by the Wilcoxon test. The correlation of immune subtypes with risk score was tested by the ANOVA analysis.

### The Gene Set Enrichment Analysis

In order to explore the underlying mechanisms, the gene set enrichment analysis (GSEA) was conducted between the high- and low-risk groups with GSEA 4.1 software to perform the Gene Ontology (GO) and Kyoto Encyclopedia of Genes and Genomes (KEGG) pathway analyses. *p*-value was adjusted by BH method.

### Cell-Cycle, Tumor Angiogenesis, and Drug Resistance Analysis

The associations of the risk score with cell cycle gene expression, tumor angiogenesis gene expression, and tumor drug resistance gene expression were tested by the Wilcoxon test.

### Chemotherapy Sensitivity Analysis

We obtained drug response data and genomic markers of sensitivity through the Genomics of Drug Sensitivity in Cancer (GDSC) database (https://www.cancerrxgene.org/). The transcriptome data and drug sensitivity data were extracted from 17 types of HCC cells in the GDSC, then Pearson’s correlation analysis was employed to explore the relationship between prognostic gene expression and antitumor drug sensitivity. The efficacy of 367 antitumor drugs was analyzed by correlation analysis (**Supplementary Table 2**).

### Validation of mRNA Expression of Prognostic Genes by Quantitative Real-Time PCR

Twenty paired HCC tissues and matched paracancerous tissue samples were recruited from patients undergoing surgery at the First Affiliated Hospital of Wenzhou Medical University. This study was reviewed and approved by the Ethical Board at the First Affiliated Hospital of Wenzhou Medical University with written informed consent from all patients. All the HCC tumor samples were confirmed by two pathological specialists independently. The samples were immediately frozen and stored in liquid nitrogen. Total RNA was isolated from HCC cancer and paracancerous tissue samples by Trizol reagent (Servicebio, Wuhan, China). Extracted RNA was then transcribed into cDNA. GAPDH was utilized to standardize the gene expression. The sequence of primers is provided in **Supplementary Table 3**. Real-time PCR analysis was performed using the StepOne Real-Time PCR System and FastStart Universal SYBR Green Master (Roche, Basel, Switzerland). Each sample was extracted in triplicate and amplified in triplicate. The gene expression was calculated using the 2^−ΔΔCt ^method.

### Validation of Protein Expression of Prognostic Genes by Immunohistochemistry

Ten paired HCC tissues and matched paracancerous tissues were utilized for immunohistochemistry (IHC) validation. This study was reviewed and approved by the Ethical Board at the First Affiliated Hospital of Wenzhou Medical University with written informed consent from all patients. Each group of HCC samples was fixed in 10% formalin at room temperature, then embedded in paraffin. Four-micrometer sections were cut from each paraffin block. The sections were then dewaxed and rehydrated. For antigen retrieval, the slices were boiled in 10 mmol/L citrate buffer (pH = 6.4) for 10 min. To inactivate the endogenous peroxidase, methanol containing 3% hydrogen peroxide was used to treat the slices. Citric acid buffer (pH = 6.0) was subsequently used to obtain optimal antigen recovery. Serum was added to block some nonspecific sites, and then the slices were placed in the incubator for half an hour at 37°C. After that, the slices were incubated with primary antibodies overnight at 4°C and secondary antibody polymer HRP for 50 min at 37°C and stained by diaminobenzidine. The cell nucleus was stained blue by hematoxylin. Lastly, the slices were sealed, subsequently observed, and photographed. The detailed primary antibody information is provided in **Supplementary Table 4**.

### Statistical Analysis

The Wilcoxon test was used to compare gene expression between cancer and paracancerous tissues. The Kaplan–Meier curve was visualized using the “survival” R package and “survminer” R package. The ssGSEA function in the “gsva” R package was used to quantify immune cell infiltration, and the scores between high- and low-risk groups were analyzed by the Wilcoxon test. The associations of the risk score with immune checkpoint gene expression, cell cycle key gene expression, and tumor angiogenesis gene expression were tested by the Wilcoxon test. The associations of the risk score with tumor drug resistance gene expression were tested by Spearman’s correlation analysis. The correlation of drug sensitivity with prognostic gene expression was explored by Pearson’s correlation. TIMER2.0 (https://timer.comp-genomics.org/) was applied to investigate the prognostic gene expression between pan-cancers and paracancerous tissues. A two-tailed *p*-value <0.05 was considered statistically significant in this study.

## Results

**Figure 1:**
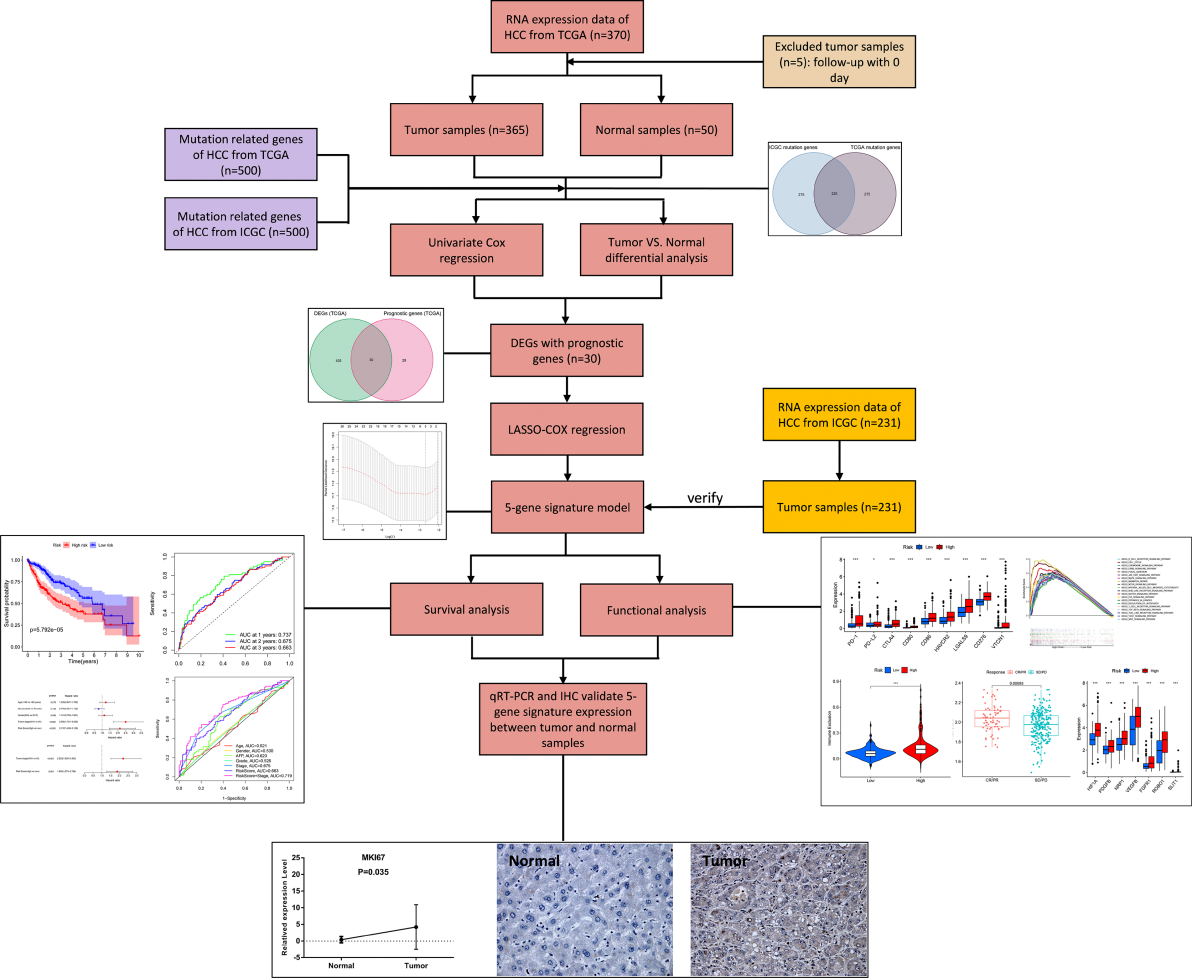
Workflow graph for this study.

The flow chart of this research is shown in **Figure 1**. A total of 370 HCC patients retrieved from TCGA database were defined as discovery dataset, and 231 HCC patients retrieved form ICGC (LIRI-JP) database were defined as validation dataset. The detailed clinical features of these patients are summarized in **Table 1**. Furthermore, an independent sample cohort including 30 matched samples were applied to validate the different expression of prognostic genes between HCC tissues and paracancerous tissues.

**Table 1:** Clinical characteristics of the HCC patients used in this study.

	TCGA-LIHC cohort	ICGC-LIRI-JP cohort
**No. of patients**	365	231
**Age (median, range)**	57 (16–90)	67 (31–89)
**Gender**		
Female	119 (32.6%)	61 (26.4%)
Male	246 (67.4%)	170 (73.6%)
**Grade**		
Grade 1	55 (15.1%)	NA
Grade 2	175 (47.9%)	NA
Grade 3	118 (32.3%)	NA
Grade 4	12 (3.3%)	NA
Unknown	5 (1.4%)	NA
**Stage**		
I	170 (46.6%)	36 (15.6%)
II	84 (23.0%)	105 (45.5%)
III	83 (22.7%)	71 (30.7%)
IV	4 (1.1%)	19 (8.2%)
Unknown	24 (6.6%)	0 (0%)
**Survival status**		
Alive	235 (64.4%)	189 (81.8%)
Deceased	130 (35.6%)	42 (18.2%)

### Landscape of Gene Mutation in HCC

**Figure 2:**
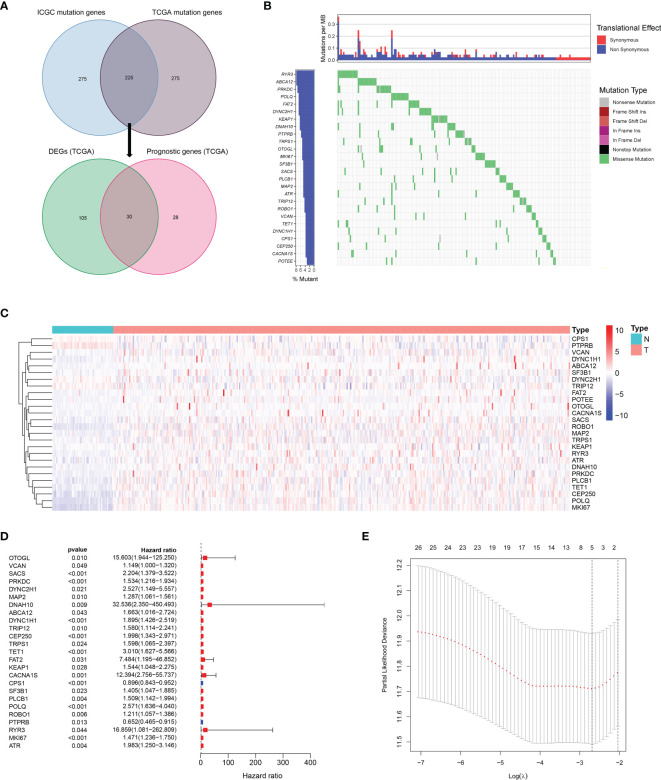
Landscapes of frequently mutated genes in HCC. **(A)** Venn diagram of mutated genes. The 225 mutated genes were from both TCGA and ICGC datasets. The 30 mutated genes with prognostic value were from TCGA dataset. **(B)** Waterfall plot displays the 26 mutated genes in HCC from TCGA dataset. The left panel presents the genes ordered by mutation frequencies. The right panel shows different types of mutation. **(C)** The heatmap of the 26 DEGs between 365 HCC tissues and 50 nontumor tissues. **(D)** Forest plot shows the 26 DEGs for OS. **(E)** LASSO coefficient spectrum of candidate genes in HCC.

A total of 225 top mutated genes were screened out from both TCGA database and the ICGC database (**Figure 2A**). Univariate Cox analysis indicated that 30 of the 135 differentially expressed mutant genes were correlated with overall survival. Among these 30 genes, the expression of 4 genes was 0 in more than 40 samples, which were eliminated. The mutation frequency ranking of the 26 mutated genes in the HCC samples from TCGA cohort is presented in **Figure 2B**. Meanwhile, the gene expression profiles of the 26 candidate mutant genes were demonstrated in the heatmap (**Figure 2C**). These candidate genes were preserved as prognostic indicators (**Figure 2D**). The TMB difference between the patients with wild gene and patients with mutation gene and the interaction network among the candidate genes are shown in **Supplementary Figure 1**. Subsequently, according to the optimal value of *λ*, the LASSO Cox regression method was employed to generate a prognostic signature model (**Figure 2E**).

### Development of Mutant Gene-Related Signature in TCGA Dataset and Validation in ICGC Dataset

**Figure 3:**
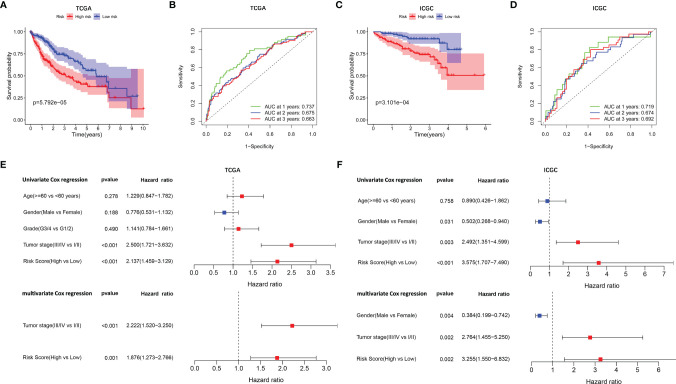
Survival analysis and independent prognostic analysis of the prognostic signature. TCGA cohort **(A, B, E)** and ICGC cohort **(C, D, F)**. **(A, C)** The Kaplan–Meier plot showed overall survival between two subgroups. **(B, D)** ROC curves of the five-gene signature for prediction of 1-, 2-, and 3-year OS. **(E, F)** Univariate and multivariate Cox regression analyses to screen prognostic factors for OS.

A prognostic model consisting of five mutated genes was established by LASSO COX regression analysis. The risk score of each patient was calculated based on the following formula: risk score = (0.0071 * expression level of MAP2) + (0.1866 * expression level of DYNC1H1) + (−0.0197 * expression level of CPS1) + (−0.0312 * expression of PTPRB) + (0.1226 * expression level of MKI67). The HCC patients were stratified into high- and low-risk groups according to median risk scores (**Supplementary Figure 2A**), and the survival status of patients in the high- and low-risk groups is shown in **Supplementary Figure 2B**. PCA and t-SNE analysis indicated that the patients in the two subgroups were distributed in discrete directions (**Supplementary Figures 2C, D**). Baseline characteristics of the patients in different risk groups were presented in **Table 2**. Kaplan–Meier curves demonstrated low-risk group had a noticeably better survival than patients in high-risk group (p = 5.792-05), indicating the risk score has an effective value of prognosis (**Figure 3A**). Also, time-dependent ROC curves were applied to assess the precision of the mutation gene signature in predicting OS of HCC patients at 1, 2, and 3 years. The area under the ROC (AUC) values of 1, 2, and 3 years were 0.737, 0.675, and 0.663, respectively (**Figure 3B**).

**Table 2:** Baseline characteristics of the patients in different risk groups.

Characteristics	TCGA-LIHC cohort			ICGC-LIRI-JP cohort		
	High risk	Low risk	*p-*value	High risk	Low risk	*p-*value
**Age**						
<60 years	86 (23.6%)	79 (21.6%)	0.433	28 (12.1%)	16 (6.9%)	0.199
≥60 years	96 (26.3%)	104 (28.5%)		99 (42.9%)	88 (38.1%)	
**Gender**						
Female	67 (18.4%)	52 (14.2%)	0.087	34 (14.8%)	27 (11.7%)	0.889
Male	115 (31.5%)	131 (35.9%)		93 (40.3%)	77 (33.3%)	
**Grade**						
G1 + G2	96 (26.3%)	134 (36.7%)	<0.001	–	–	
G3 + G4	83 (22.7%)	47 (12.9%)		–	–	
Unknown	3 (0.8%)	2 (0.5%)		–	–	
**Stage**						
I + II	112 (30.7%)	142 (38.9%)	0.001	67 (29.0%)	74 (32.0%)	0.004
III + IV	57 (15.6%)	30 (8.2%)		60 (26.0%)	30 (13.0%)	
Unknown	13 (3.6%)	11 (3.0%)		0 (0.0%)	0 (0.0%)	

To verify the stability of the prognostic model constructed from TCGA dataset, the patients from the ICGC dataset were also categorized into high- and low-risk groups based on the median value from TCGA (**Supplementary Figure 2E**). Likewise, the outcomes of patients were also poorer in high-risk group compared with low-risk group (**Supplementary Figure 2F**). PCA and t-SNE analysis confirmed a discrete distribution of patients in two risk groups (**Supplementary Figures 2G, H**). Kaplan–Meier curves similarly showed that patients in the high-risk group had an inferior OS compared with their counterparts (*p* = 3.101e−04, **Figure 3C**). Furthermore, the AUC of the mutation-related gene signature reached 0.719 at 1 year, 0.674 at 2 years, and 0.692 at 3 years, respectively (**Figure 3D**).

### Independent Prognostic Value of the Prognostic Signature Model

**Figure 4:**
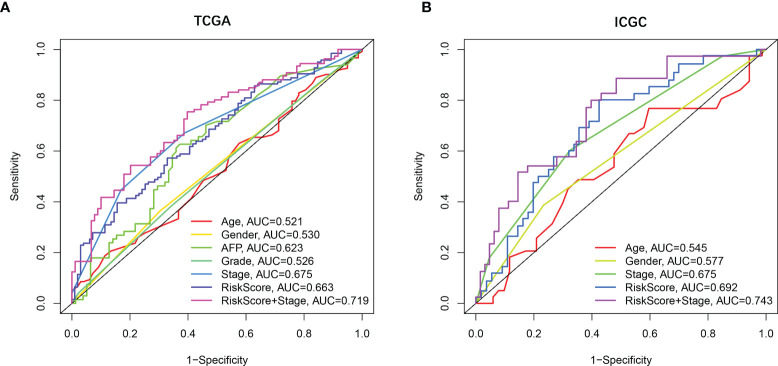
The prognostic value validated by multi-index ROC curves. **(A)** TCGA cohort, *n* = 365, curves in different colors represent time-dependent ROC of age, gender, AFP, grade, stage, risk score, and risk score with stage variables, respectively. **(B)** ICGC cohort, *n* = 231, curves in different colors represent time-dependent ROC of age, gender, stage, risk score, and risk score with stage variables, respectively.

Univariate and multivariate Cox regression analyses were employed to evaluate the prognostic value of the risk scores. In the univariate Cox regression analysis, the risk score was significantly correlated with OS in both TCGA and the ICGC dataset (TCGA: HR = 2.137, 95% CI = 1.459–3.129, *p* < 0.001, **Figure 3E**; ICGC: HR = 3.575, 95% CI = 1.707–7.490, *p* < 0.001, **Figure 3F**). The multivariate Cox regression analysis confirmed the risk score was also an independent predictor of OS (TCGA cohort: HR = 1.876, 95% CI = 1.273–2.766, *p* = 0.001; ICGC cohort: HR = 3.255, 95% CI = 1.550–6.832, *p* = 0.002). In addition, the prognostic performance of risk score combined with tumor stage was evaluated by time-dependent ROC curve, which exhibited a higher prognostic value for 3-year OS than tumor stage, wherever in TCGA set (stage AUC = 0.675, risk score + stage AUC = 0.719, **Figure 4A**) and ICGC cohort (stage AUC = 0.675, risk score + stage AUC = 0.743, **Figure 4B**).

### Prognostic Model Risk Score and Clinical Features

The correlation between the risk score based on the signature of mutation gene and several clinical features was assessed (**Supplementary Figure 3**). The results revealed that high-risk score was significantly associated with grades G3–G4 and stages III–IV, while low-risk score was significantly associated with grades G1–G2 and stages I–II (*p* < 0.001, **Supplementary Figures 3C, D**). Consistently, the ICGC dataset exhibited similar results (No data about the tumor grade of HCC in the ICGC dataset, *p* < 0.001, **Supplementary Figure 3G**).

The expression of MAP2, DYNC1H1, and MKI67 was obviously higher expressed in tumor tissues, while CPS1 and PTPRB were downregulated in tumor tissues compared with normal tissues in TCGA dataset (**Supplementary Figures 4A–E**). Interestingly, Kaplan–Meier curves for individual predictive of each prognostic gene demonstrated that high expression of MAP2, DYNC1H1, and MKI67 was infaust for OS, while high expression of CPS1 and PTPRB were helpful for prognosis (**Supplementary Figures 4F, G**). Furthermore, the correlation between prognostic gene expression and clinical characteristics of HCC patients indicated that DYNC1H1 and MKI67 were significantly upregulated in tumor grades G3–G4 and tumor stages III–IV compared with tumor grades G1–G2 and tumor stages I–II, while CPS1 expression was downregulated in tumor grades G3–G4 and tumor stages III–IV (*p* < 0.05, **Supplementary Figures 5C, D**).

### Immune Status Between High-Risk and Low-Risk Groups

**Figure 5:**
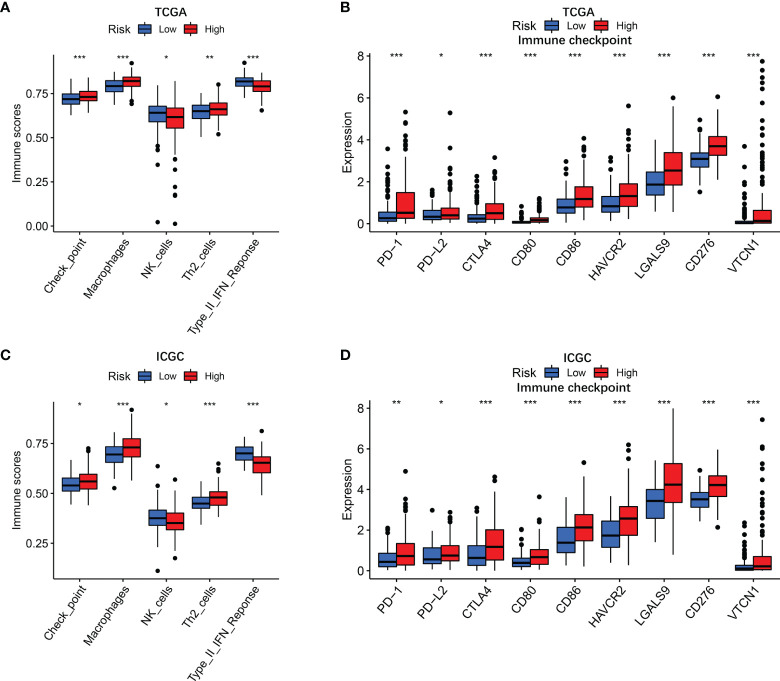
Immune status in different risk groups. TCGA cohort **(A, B)**, ICGC cohort **(C, D)**. **(A, C)** The relative enrichment of 5 immune-related risk terms in different risk groups. **(B, E)** The associations of risk score with PD-L2 expression and galectin-9 expression. **(B, D)** Expression levels of immune checkpoints in different risk groups. P values were showed as: ns, not significant; *P < 0.05; **P < 0.01; ***P < 0.001.

To further clarify the mechanism underlying the functions of the risk score, the differences in immune function and immune cell infiltration between the two groups are presented in **Figure 5**. The ssGSEA results showed that the abundance of Check point, Macrophages, and Th2 cells were significantly elevated in the high-risk group. On the contrary, NK cells and type II IFN response were significantly downregulated in the high-risk group. Considering that the expression levels of immune checkpoints serve as important indicators for individualized immunotherapy, the present study identified that the expression of PD-1, PD-L2, CTLA4, CD80, CD86, HAVCR2, LAGLS9, CD274, and VTCN1 was higher in high-risk patients compared with their low-risk counterpart.

**Figure 6:**
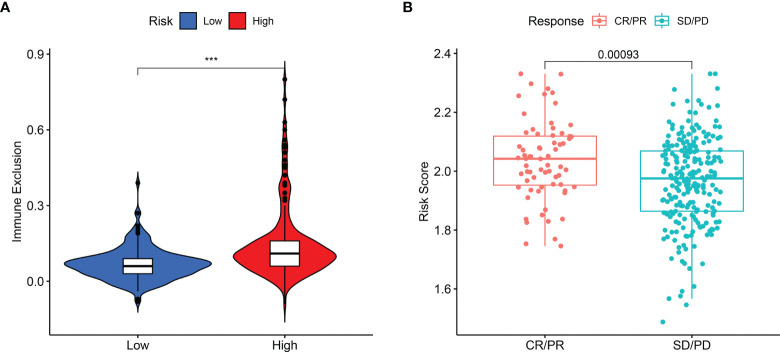
The difference in immune exclusion between high- and low-risk groups and the distribution of risk scores in immunotherapy response group. **(A)** Immune exclusion levels in two subgroups. **(B)** Distribution of risk scores in the distinct anti‐PD‐1 clinical response group. P values were showed as: ns, not significant; *P < 0.05; **P < 0.01; ***P < 0.001.

The present study revealed that the capability of immune exclusion in HCC was significantly higher in high-risk group compared with low-risk group (*p* < 0.001, **Figure 6A**). When the prognostic model was applied to the IMvigor210 study, a clinical study to evaluate the safety and efficacy of PD-L1 monoclonal antibody in the treatment of metastatic urothelial carcinoma, the risk score of patients with complete response (CR) or partial response (PR) was higher than that of patients with stable disease (SD) or progressive disease (PD) (**Figure 6B**, *p* = 0.00093).

Thorsson et al. have identified a pan-cancer immune classification which encompasses almost all human malignancies and consists of six immune subtypes ranking from tumor promoting to tumor inhibiting, respectively: C1 (wound healing), C2 (INF-r dominant), C3 (inflammatory), C4 (lymphocyte depleted), C5 (immunologically quiet), and C6 (TGF-β dominant) (24). No patient sample belonged to C5 immune subtype in HCC and only one sample belonged to C6 immune subtype, which was eliminated. In the present study, high-risk score was significantly associated with C1 and C2, while low-risk score was significantly associated with C3 and C4 (**Supplementary Figure 6A**). In addition, the correlation of each prognostic gene with immune subtype showed high expression of DYNC1H1 and MKI67 had a significant association with C1 and C2 immune subtypes, while high expression of CPS1 and PTPRB was significantly associated with C3 and C4 immune subtypes (**Supplementary Figure 6B**).

### Gene Ontology and Kyoto Encyclopedia of Genes and Genomes Pathway Analyses by Gene Set Enrichment Analysis

**Figure 7:**
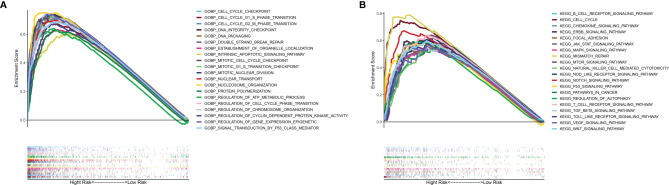
GSEA of biological functions and pathways. **(A)** Gene Ontology. **(B)** Kyoto Encyclopedia of Genes and Genomes.

To elucidate the potential mechanism of the mutation-related classifier on HCC, GSEA was applied to compare the high-risk group and low-risk group. Based on the mutation-related risk groups, GSEA revealed that cell cycle-related biological processes, including cell cycle checkpoint, cell cycle G1 S phase transition, cell cycle G2 M phase transition, mitotic cell cycle checkpoint, and regulation of cell cycle phase transition, were significantly enriched in the high-risk group (**Figure 7A**; **Supplementary Figure 7**). Also, KEGG pathway analysis indicated that cell cycle pathways, immune-related pathways (B-cell receptor and T-cell receptor signaling pathway), and tumor-related pathways (pathway in cancer and VEGF, JAK-STAT, mTOR, MAPK, WNT, and NOTCH signaling pathways) were significantly enriched in the high-risk group (**Figure 7B**; **Supplementary Figure 8**).

### Cell Cycle Gene and Tumor Angiogenesis Gene Analysis

**Figure 8:**
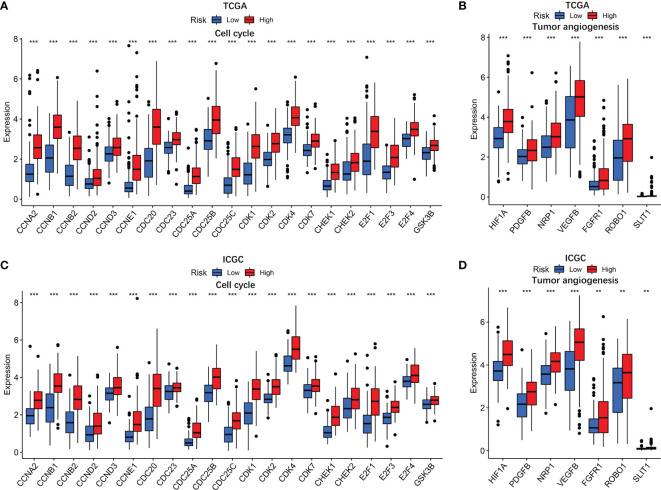
The associations of risk score with cell cycle genes and tumor angiogenesis genes. TCGA cohort **(A, B)** and ICGC cohort **(C, D)**. P values were showed as: ns, not significant; *P < 0.05; **P < 0.01; ***P < 0.001.

GO and KEGG analyses revealed that cell cycle biological process and cell cycle pathways were enriched in the high-risk groups. The correlation analysis was then conducted to analyze the relation of risk score with cell cycle genes. The results indicated that cell cycle genes (CCNA2, CCNB1, CCNB2, CCND2, CCND3, CCNE1, CDC20, CDC23, CDC25A, CDC25B, CDC25C, CDK1, CDK2, CDK4, CDK7, CHEK1, CHEK2, E2F1, E2F3, E2F4, and GSK3B) had substantially higher expression in the high-risk group (**Figure 8A**). In addition, we also analyzed tumor angiogenesis genes, and the results showed that seven tumor angiogenesis genes (HIF1A, PDGFB, NRP1, VEGFB, FGFR1, ROBO1, and SLIT1) had substantially higher expression in the high-risk group compared with the low-risk group (**Figure 8B**). Corresponding results for the validation cohort (**Figures 8C, D**) were similar to the training cohort.

### Multidrug Resistance Gene and Anti-tumor Drug Resistance Analysis

**Figure 9:**
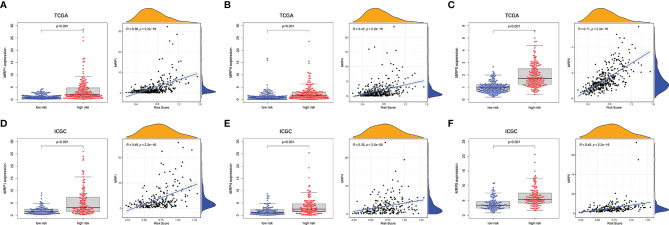
The associations between tumor drug resistance genes and risk score. TCGA cohort **(A–C)** and ICGC cohort **(D–F)**.

To evaluate the prognostic model for HCC treatment, we further explored correlations of the prognostic genes with the multidrug resistance-related genes. As demonstrated in **Figure 9**, multidrug resistance protein 1 (MRP1), MRP4, and MRP5 were obviously elevated in the high-risk group and they all had a strong positive correlation with the risk score. Moreover, the correlation of drug resistance of HCC cells with prognostic genes revealed that the prognostic genes were significantly associated with resistance of HCC cells to chemotherapy drugs (**Supplementary Figure 9**). For example, expression of MAP2, DYNC1H1, and MKI67 were positively correlated with drug resistance of HCC cells to trametinib, bleomycin, methotrexate, and talazoparib. By contrast, there was a significantly negative correlation of the expression of CPS1 and PTPRB with resistance of HCC cells to antitumor drugs such as palbociclib, talazoparib, and bosutinib.

### Experimental Verification of the Expression of the Five Prognostic Genes

**Figure 10:**
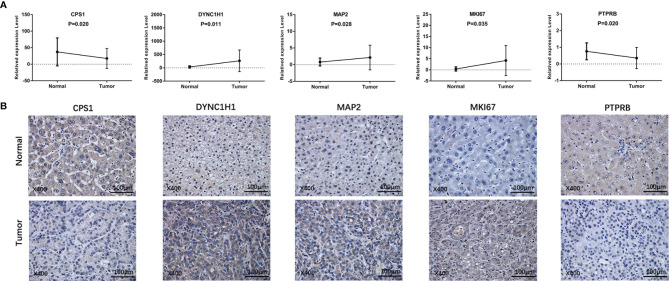
Prognostic gene expression between HCC and paracancerous tissues was validated by independent sample cohorts. **(A)** qRT-RCR. **(B)** IHC.

To further validate our findings, we measured the expression of the five prognostic genes in HCC tissues and paracancerous tissues. As shown in **Figure 10A**, quantitative real-time PCR (qRT-PCR) exhibited that expression of CPS1 and PTPRB was downregulated in tumor tissues compared with paracancerous tissues, while MAP2, DYNC1H1, and MKI67 were overexpressed in tumor tissues. Consistently, IHC staining showed similar results as qRT-PCR (**Figure 10B**). The above results were consistent with the RNA sequencing expression of 5 mutation-related prognostic genes in TCGA dataset (**Supplementary Figure 4**).

### The Expression Analysis of the Five Prognostic Genes in Pan-Cancers and Para-cancerous Tissues

The expression of five prognostic genes in other types of cancer samples was analyzed, and we found that five prognostic genes had differential expression in most of other cancers and paracancerous tissues (*p* < 0.05) (**Supplementary Figure 10**).

## Discussion

HCC is known as a high incidence and mortality malignancy globally, which is the major contributor to the worldwide cancer burden. The pathology of HCC is complex, presenting a challenge in the development of effective treatments for HCC. Surgical resection has been the main treatment for HCC in recent decades. Although the treatment of HCC such as chemotherapy, molecular-targeted therapies, microwave ablation, interventional therapy, and liver transplantation has evolved considerably, the patients with HCC continue to have a dismal prognosis. AFP is the widely used as a serum biomarker for evaluating prognosis and is incorporated into several staging systems. However, the application of AFP in the diagnosis and prognosis prediction of HCC is limited due to its low sensitivity (25). Thus, screening novel biomarkers and prognostic predictors of HCC remains an urgent need in order to reduce the mortality and improve outcomes.

In this study, a mutation-related gene signature was constructed by integrating the transcriptomic data and clinical information of patients with HCC. LASSO Cox regression method identified 5 mutated prognostic genes to construct a prognostic model, including CPS1, DYNC1H1, MAP2, MKI67, and PTPRB. In addition to the prognostic model based on traditional prognostic markers, genomics and bioinformatics have made it possible to identify prognostic gene signatures. Some research confirmed that a number of gene signatures have recently been developed to predict outcomes of patients with HCC (21, 22, 26). However, these gene signatures could not predict the immune status of the body and drug resistance of cancer cells. Compared with these models, the prognostic signature in this study can distinguish the immune status and risk score was strongly associated with cell cycle and drug resistance. Moreover, the independent sample cohort was employed to validate the differential expression of prognostic genes. Among these genes, MKI67, MAP2, and DYNC1H1 are well-known genes in regulating cell cycle and cell divisions. Antigen Ki-67, also known as MKI67, which is a prototypic cell cycle nuclear protein expressed in G1, S, and G2 and peaking at M phase (27). MKI67 is strictly associated with cell proliferation and is widely used as a prognostic marker in several tumors (28, 29), including identifying patients who are at greatest risk for postsurgical recurrence of HCC (30). MAP2 has been found to perform a range of functions in regulating microtubule cytoskeleton dynamics (31) and DYNC1H1 carries organelles and key signals from distal sites to the cell body (32). Thus, MAP2 and DYNC1H1 are closely related to cell cycle. However, few studies have focused on the role of MAP2 in tumorigenesis and development. A critical factor in the progression of hepatocytes into malignant HCC cells may be metabolizing reprogramming through decreased CPS1 RNA expression and hypermethylation of CPS1 to transform cells dedicated to normal body support function into cells that only support their own growth and division (12). Recently, it has been found that PTPRB may function as a tumor suppressor in tumorigenesis and development (33). Most of these genes have not been proved to be directly related to HCC, and the underlying mechanisms are worthy of further theoretical and experimental investigations.

To elucidate the potential correlation, ssGSEA algorithm was adopted for quantifying the activities or abundances of several types of immune pathways and immune cells in every HCC specimen. Due to the importance in immune invasion, tumor-associated macrophage has been reported to closely associate with poor prognosis in patients with HCC (34, 35). Also, antitumor immunity was impaired more seriously in the high-risk group as the reduced proportion of NK cells and the decreased activity of type II IFN response. Additionally, acquired properties enable tumor cells to activate immune checkpoint pathways that evade immunosurveillance and suppress immune system responses (36). The abnormal expression of immune checkpoint markers may lead to the occurrence or progression of many diseases (37). Immune checkpoint blockades recently gain substantial ground, which have become the benchmark treatment for cancer therapy (38). The positive correlation between risk score and the levels of immune checkpoints was demonstrated, suggesting that HCC in high-risk patients has a stronger ability to evade immune monitoring and suppress antitumor immunity. In this perspective, the predictive model may offer insights for accelerating the pace of individualized cancer immunotherapy. Furthermore, in the present study, risk scores appeared to positively associate with immune exclusion, a process whereby tumor lacks T-cell infiltration (39–41). Interestingly, in the IMvigor210 cohort, the high-risk group was linked to a stronger response to anti-PD-L1 immunotherapy, which may be explained by abnormally elevated immune checkpoint gene expression, indicating that the prognostic model can distinguish the efficacy of immunotherapy in urothelial carcinoma. The robust predictive capability of the model may also play a role in the efficacy evaluation of immunotherapy for HCC in the future.

Deregulated cell proliferation is the most fundamental biological feature of tumors, which is based on the disturbed cell cycle regulation (42). Although tumorigenesis involves various processes that also provide targets for cancer treatment, in almost all instances, deregulated cell proliferation and inhibited cell death together provide the underlying platform for tumorigenesis and progression (43). Identification of the definite mechanism of such pivotal steps in tumor progression and development of therapies that directly attack these points of convergence are the major challenges before the research community. In this study, cell cycle-related biological processes were observed to be significantly enriched in the high-risk group and key genes in cell cycle was substantially higher expressed in the high-risk group. CDK2 was reported to have the ability of accelerating S phase initiation in cell cycle (44) and is related with the tumorigenesis of HCC (45). Unlike CDK2, the enzymatic activities of CDK4 are governed by D-type cyclins, which respond to various extracellular signals. Rapidly emerging data with selective CDK4/6 inhibitor have corroborated these kinases in cell cycle as antitumor drug targets and long-standing preclinical predictions (46). Abnormal expression of cyclin E was detected in various high-grade malignant cells (47–51), which is a key link in the chemotherapy resistance mechanism of various tumor cells and may be used as a potential treatment target to reverse or reduce antitumor drug resistance in cancer therapy (52). CDC25A, as the oncogene, is overexpressed in a variety of human malignancies and could be used as an independent prognostic marker for HCC (53). Therefore, facilitating the division of tumor cells may be the pivotal approach of high-risk score to influence the prognosis of HCC patients.

Chemoresistance remains the major unmet obstacle encountered during the clinical therapy of HCC. Notably upregulated MRPs that are famous for their function in actively extruding chemotherapeutic substrates are essential components of multidrug resistance (54). Interestingly, the risk score was significantly positively correlated with expression of MRPs. Modulating the function of MRPs in HCC to resensitize chemotherapeutic drugs may have great prospects of utilization. However, although there is a correlation between prognostic gene and the drug resistance of HCC patients to some antitumor drugs, the link between high-risk group and increased drug resistance is quite complex. Thus, the direct relationship between prognostic genes and antitumor drugs cannot be determined, which needs further experimental proof.

## Conclusion

Our study provides a comprehensive and systematic characterization of the profiles of the novel prognostic model constructed with 5 mutated genes retrieved from a public database. Taken together, our work will greatly contribute to uncover the role of prognostic model in the prognosis of HCC. The underlying mechanism between the model and tumor immunity in HCC remains poorly understood and warrants further investigation.

## Limitation

In this study, there is a lack of independent sample set to further verify the predictive power of mutation gene signature on the survival of HCC patients. In addition, the effect of mutation-gene signature on immune function of HCC patients needs to be further verified by independent samples.

## Data Availability Statement

The original contributions presented in the study are included in the article/**Supplementary Material**. Further inquiries can be directed to the corresponding authors.

## Ethics Statement

The studies involving human participants were reviewed and approved by the Ethical Board at the First Affiliated Hospital of Wenzhou Medical University. The patients/participants provided their written informed consent to participate in this study. Written informed consent was obtained from the individual(s) for the publication of any potentially identifiable images or data included in this article.

## Author Contributions

FY and ZC designed the current study. XS, YZ, LWZ, LYZ, JX, and DM collected and analyzed the data. ZL and QX performed the experiment. ZL drafted the manuscript. FY and ZC supervised the study. All authors have verified the underlying data and agreed to submit it in its current form for consideration for publication.

## Funding

This research was funded by the National Natural Science Foundation of China (No. 81970527) and the Zhejiang Provincial Natural Science Foundation of China (No. LY19H030005). The funders had no role in the study design, data collection, data analysis, interpretation, and writing of the manuscript.

## Conflict of Interest

The authors declare that the research was conducted in the absence of any commercial or financial relationships that could be construed as a potential conflict of interest.

## Publisher’s Note

All claims expressed in this article are solely those of the authors and do not necessarily represent those of their affiliated organizations, or those of the publisher, the editors and the reviewers. Any product that may be evaluated in this article, or claim that may be made by its manufacturer, is not guaranteed or endorsed by the publisher.

## Supplementary Material

Supplementary Table 1 The top mutant genes of HCC retrieved from TCGA and ICGC databases and corresponding mutation number.

Supplementary Table 2 The anti-tumor drugs with anti-tumor targets.

Supplementary Table 3 The primer sequences of the five prognostic genes.

Supplementary Table 4 The detailed information of primary antibodies used in the study.

Supplementary Table 5 The correlation between the prognostic gene’s expression and the anti-tumor drugs resistance.

Supplementary Figure 1 The TMB difference between the patients with wild gene and patients with mutation gene and the correlation between the candidate genes. **(A)** Patients are grouped according to the gene wild type and mutation type, and then the TMB difference between each wild type group and mutation group was calculated. **(B)** The correlation between the 26 candidate genes. P values were showed as: ns, not significant; *P< 0.05; **P< 0.01; ***P< 0.001.

Supplementary Figure 2 Survival status of High- and low-risk patients. TCGA cohort **(A-D)**, ICGC cohort **(E-H)**. **(A, B, E, F)** Risk score distribution, patients’ survival status and time for high‐ and low‐risk groups in the entire set. **(C, D, G, H)** PCA and t-SNE analyses showed distinct clustering in high-risk patients and low-risk patients.

Supplementary Figure 3 The risk score in different groups stratified by clinical characteristics.

Supplementary Figure 4 The prognostic gene expression between HCC and para-cancerous tissues in the TCGA cohort and Kaplan–Meier survival curve of each prognostic gene for the patients with HCC in TCGA dataset.

Supplementary Figure 5 The prognostic gene expression in different groups stratified by clinical characteristics.

Supplementary Figure 6 The risk score and prognostic gene expression in different immune infiltration subtypes.

Supplementary Figure 7 The GSEA diagram of GO biological processes.

Supplementary Figure 8 The GSEA diagram of KEGG pathways.

Supplementary Figure 9 The correlation of prognostic gene expression with drug resistance. MAP2. **(B)** DYNC1H1. **(C)** CPS1. **(D)** PTPRB. **(E)** MKI67.

Supplementary Figure 10 Prognostic gene expression between pan-cancers and para-cancerous tissues.
