# Supplementary material for: Comprehensive Analysis Identified Mutation-Gene Signature Impacts the Prognosis Through Immune Function in Hepatocellular Carcinoma
Source: Front Oncol. 2022 Mar 4;12:748557. doi: 10.3389/fonc.2022.748557 (PMC8931204; doi:10.3389/fonc.2022.748557)
Supplement: Supplementary file 1 [file DataSheet_1.docx]

#The different expression of mutation genes between HCC and adjacent normal tissues

library(limma)

inputFile="input.txt"

fdrFilter=0.05

logFCfilter=0

for(i in row.names(data)){

geneName=unlist(strsplit(i,"\\|",))[1]

geneName=gsub("\\/", "_", geneName)

rt=rbind(expression=data[i,],grade=grade)

rt=as.matrix(t(rt))

wilcoxTest<-wilcox.test(expression ~ grade, data=rt)

conGeneMeans=mean(data[i,1:conNum])

treatGeneMeans=mean(data[i,(conNum+1):ncol(data)])

logFC=log2(treatGeneMeans)-log2(conGeneMeans)

pvalue=wilcoxTest$p.value

conMed=median(data[i,1:conNum])

treatMed=median(data[i,(conNum+1):ncol(data)])

diffMed=treatMed-conMed

if( ((logFC>0) & (diffMed>0)) | ((logFC<0) & (diffMed<0)) ){

outTab=rbind(outTab,cbind(gene=i,conMean=conGeneMeans,treatMean=treatGeneMeans,logFC=logFC,pValue=pvalue))

}

}

pValue=outTab[,"pValue"]

fdr=p.adjust(as.numeric(as.vector(pValue)),method="fdr")

outTab=cbind(outTab,fdr=fdr)

write.table(outTab,file="all.xls",sep="\t",row.names=F,quote=F)

outDiff=outTab[( abs(as.numeric(as.vector(outTab$logFC)))>logFCfilter & as.numeric(as.vector(outTab$fdr))<fdrFilter),]

write.table(outDiff,file="diff.xls",sep="\t",row.names=F,quote=F)

outDiff=outTab[( abs(as.numeric(as.vector(outTab$logFC)))>logFCfilter & as.numeric(as.vector(outTab$fdr))<fdrFilter),]

write.table(outDiff,file="diff.txt",sep="\t",row.names=F,quote=F)

up=outTab[(as.numeric(as.vector(outTab$logFC))>logFCfilter & as.numeric(as.vector(outTab$fdr))<fdrFilter),]

write.table(up,file="up.txt",sep="\t",row.names=F,quote=F)

down=outTab[(as.numeric(as.vector(outTab$logFC))< -logFCfilter & as.numeric(as.vector(outTab$fdr))<fdrFilter),]

write.table(down,file="down.txt",sep="\t",row.names=F,quote=F)

heatmap=rbind(ID=colnames(data[as.vector(outDiff[,1]),]),data[as.vector(outDiff[,1]),])

write.table(heatmap,file="tcgaDiffRBPexp.txt",sep="\t",col.names=F,quote=F)

#LASSO

library("glmnet")

library("survival")

coxSigFile="input.txt"

geneFile="gene.txt"

rt=read.table(coxSigFile,header=T,sep="\t",row.names=1)

geneRT=read.table(geneFile,header=F,sep="\t",check.names=F)

rt=rt[,c("futime","fustat",as.vector(geneRT[,1]))]

rt$futime=rt$futime/365

x=as.matrix(rt[,c(3:ncol(rt))])

y=data.matrix(Surv(rt$futime,rt$fustat))

fit=glmnet(x, y, family = "cox", maxit = 1000)

pdf("lasso.lambda.pdf")

plot(fit, xvar = "lambda", label = TRUE)

dev.off()

cvfit=cv.glmnet(x, y, family="cox", maxit = 1000)

pdf("lasso.cvfit.pdf")

plot(cvfit)

abline(v=log(c(cvfit$lambda.min,cvfit$lambda.1se)),lty="dashed")

dev.off()

coef=coef(fit, s = cvfit$lambda.min)

index=which(coef != 0)

actCoef=coef[index]

lassoGene=row.names(coef)[index]

geneCoef=cbind(Gene=lassoGene,Coef=actCoef)

write.table(geneCoef,file="geneCoef.txt",sep="\t",quote=F,row.names=F)

#Survival curve

library(survival)

library(survminer)

bioSurvival=function(inputFile=null,outFile=null){

rt=read.table(inputFile,header=T,sep="\t")

diff=survdiff(Surv(futime, fustat) ~risk,data = rt)

pValue=1-pchisq(diff$chisq,df=1)

pValue=signif(pValue,4)

pValue=format(pValue, scientific = TRUE)

fit <- survfit(Surv(futime, fustat) ~ risk, data = rt)

surPlot=ggsurvplot(fit,

data=rt,

conf.int=TRUE,

pval=paste0("p=",pValue),

pval.size=5,

legend.labs=c("High risk", "Low risk"),

legend.title="Risk",

xlab="Time(years)",

break.time.by = 1,

risk.table.title="",

palette=c("red", "blue"),

risk.table=F,

risk.table.height=.25)

pdf(file=outFile,onefile = FALSE,width = 5,height =4.5)

print(surPlot)

dev.off()

}

bioSurvival(inputFile="input.txt",outFile="survival.pdf")

#ROC curve

library(survival)

library(survminer)

library(timeROC)

bioROC=function(inputFile=null,rocFile=null){

rt=read.table(inputFile,header=T,sep="\t")

ROC_rt=timeROC(T=rt$futime,delta=rt$fustat,

marker=rt$riskScore,cause=1,

weighting='aalen',

times=c(1,2,3),ROC=TRUE)

pdf(file=rocFile,width=5,height=5)

plot(ROC_rt,time=1,col='green',title=FALSE,lwd=2)

plot(ROC_rt,time=2,col='blue',add=TRUE,title=FALSE,lwd=2)

plot(ROC_rt,time=3,col='red',add=TRUE,title=FALSE,lwd=2)

legend('bottomright',

c(paste0('AUC at 1 years: ',sprintf("%.03f",ROC_rt$AUC[1])),

paste0('AUC at 2 years: ',sprintf("%.03f",ROC_rt$AUC[2])),

paste0('AUC at 3 years: ',sprintf("%.03f",ROC_rt$AUC[3]))),

col=c("green",'blue','red'),lwd=2,bty = 'n')

dev.off()

}

bioROC(inputFile="input.txt",rocFile="ROC.pdf")

#PCA and t-SNE analysis

library(Rtsne)

library(ggplot2)

rt=read.table(inputFile,sep="\t",header=T,row.names=1,check.names=F)

data=rt[c(3:(ncol(rt)-2))]

risk=rt[,"risk"]

data.pca=prcomp(data, scale. = TRUE)

pcaPredict=predict(data.pca)

PCA = data.frame(PC1 = pcaPredict[,1], PC2 = pcaPredict[,2],risk=risk)

pdf(file=pcaFile, height=3.5, width=5.3)

p=ggplot(data = PCA, aes(PC1, PC2)) + geom_point(aes(color = risk)) +

scale_colour_manual(name="Risk", values =c("red", "blue"))+

theme_bw()+

theme(plot.margin=unit(rep(1.5,4),'lines'))+

theme(panel.grid.major = element_blank(), panel.grid.minor = element_blank())

print(p)

dev.off()

tsneOut=Rtsne(data, dims=2, perplexity=10, verbose=F, max_iter=500,check_duplicates=F)

tsne=data.frame(tSNE1 = tsneOut$Y[,1], tSNE2 = tsneOut$Y[,2],risk=risk)

pdf(file=tsneFile, height=3.5, width=5.3)

p=ggplot(data = tsne, aes(tSNE1, tSNE2)) + geom_point(aes(color = risk)) +

scale_colour_manual(name="Risk", values =c("red", "blue"))+

theme_bw()+

theme(plot.margin=unit(rep(1.5,4),'lines'))+

theme(panel.grid.major = element_blank(), panel.grid.minor = element_blank())

print(p)

dev.off()

}

bioPCA(inputFile="input.txt", pcaFile="PCA.pdf", tsneFile="t-SNE.pdf")

#Univariate and multivariate regression analysis

library(survival)

risk=read.table("input.txt",header=T,sep="\t",check.names=F,row.names=1)

cli=read.table("clinical.txt",sep="\t",check.names=F,header=T,row.names=1)

sameSample=intersect(row.names(cli),row.names(risk))

risk=risk[sameSample,]

cli=cli[sameSample,]

rt=cbind(futime=risk[,1],fustat=risk[,2],cli)

uniTab=data.frame()

for(i in colnames(rt[,3:ncol(rt)])){

cox <- coxph(Surv(futime, fustat) ~ rt[,i], data = rt)

coxSummary = summary(cox)

uniTab=rbind(uniTab,

cbind(id=i,

HR=coxSummary$conf.int[,"exp(coef)"],

HR.95L=coxSummary$conf.int[,"lower .95"],

HR.95H=coxSummary$conf.int[,"upper .95"],

pvalue=coxSummary$coefficients[,"Pr(>|z|)"])

)

}

write.table(uniTab,file="train.uniCox.txt",sep="\t",row.names=F,quote=F)

uniTab=uniTab[as.numeric(uniTab[,"pvalue"])<0.05,]

rt1=rt[,c("futime","fustat",as.vector(uniTab[,"id"]))]

multiCox=coxph(Surv(futime, fustat) ~ ., data = rt1)

multiCoxSum=summary(multiCox)

multiTab=data.frame()

multiTab=cbind(

coef=multiCoxSum$coefficients[,"coef"],

HR=multiCoxSum$conf.int[,"exp(coef)"],

HR.95L=multiCoxSum$conf.int[,"lower .95"],

HR.95H=multiCoxSum$conf.int[,"upper .95"],

pvalue=multiCoxSum$coefficients[,"Pr(>|z|)"])

multiTab=cbind(id=row.names(multiTab),multiTab)

write.table(multiTab,file="train.multiCox.txt",sep="\t",row.names=F,quote=F)

bioForest=function(coxFile=null,forestFile=null,forestCol=null){

rt <- read.table(coxFile,header=T,sep="\t",row.names=1,check.names=F)

gene <- rownames(rt)

hr <- sprintf("%.3f",rt$"HR")

hrLow <- sprintf("%.3f",rt$"HR.95L")

hrHigh <- sprintf("%.3f",rt$"HR.95H")

Hazard.ratio <- paste0(hr,"(",hrLow,"-",hrHigh,")")

pVal <- ifelse(rt$pvalue<0.001, "<0.001", sprintf("%.3f", rt$pvalue))

pdf(file=forestFile, width = 9,height = 3.5)

n <- nrow(rt)

nRow <- n+1

ylim <- c(1,nRow)

layout(matrix(c(1,2),nc=2),width=c(3,2.5))

xlim = c(0,3)

par(mar=c(4,2.5,2,1))

plot(1,xlim=xlim,ylim=ylim,type="n",axes=F,xlab="",ylab="")

text.cex=0.8

text(0,n:1,gene,adj=0,cex=text.cex)

text(2-0.5*0.2,n:1,pVal,adj=1,cex=text.cex);text(2-0.5*0.2,n+1,'pvalue',cex=text.cex,font=2,adj=1)

text(3,n:1,Hazard.ratio,adj=1,cex=text.cex);text(3,n+1,'Hazard ratio',cex=text.cex,font=2,adj=1,)

par(mar=c(4,1,2,1),mgp=c(2,0.5,0))

xlim = c(0,max(as.numeric(hrLow),as.numeric(hrHigh)))

plot(1,xlim=xlim,ylim=ylim,type="n",axes=F,ylab="",xaxs="i",xlab="Hazard ratio")

arrows(as.numeric(hrLow),n:1,as.numeric(hrHigh),n:1,angle=90,code=3,length=0.05,col="black",lwd=1)

abline(v=1,col="black",lty=2,lwd=1)

boxcolor = ifelse(as.numeric(hr) > 1,'red', 'blue')

points(as.numeric(hr), n:1, pch = 15, col = boxcolor, cex=1.3)

axis(1)

dev.off()

}

bioForest(coxFile="uniCox.txt",forestFile="uniForest.pdf")

bioForest(coxFile="multiCox.txt",forestFile="multiForest.pdf")

#MultiROC curve

library(survival)

library(survminer)

library(timeROC)

inputFile="input.txt"

outFile="ROC.pdf"

rt=read.table(inputFile, header=T, sep="\t", check.names=F, row.names=1)

bioCol=rainbow(ncol(rt)-2)

aucText=c()

pdf(file=outFile,width=6,height=6)

i=3

ROC_rt=timeROC(T=rt$futime,delta=rt$fustat,marker=rt[,i],cause=1,weighting='aalen',times=c(3),ROC=TRUE)

plot(ROC_rt,time=3,col=bioCol[i-2],title=FALSE,lwd=2)

aucText=c(paste0(colnames(rt)[i],", AUC=",sprintf("%.3f",ROC_rt$AUC[2])))

abline(0,1)

for(i in 4:ncol(rt)){

ROC_rt=timeROC(T=rt$futime,delta=rt$fustat,marker=rt[,i],cause=1,weighting='aalen',times=c(3),ROC=TRUE)

plot(ROC_rt,time=3,col=bioCol[i-2],title=FALSE,lwd=2,add=TRUE)

aucText=c(aucText,paste0(colnames(rt)[i],", AUC=",sprintf("%.3f",ROC_rt$AUC[2])))

}

legend("bottomright", aucText,lwd=2,bty="n",col=bioCol[1:(ncol(rt)-2)])

dev.off()

#The different expression of genes between high-risk and low-risk groups

library(limma)

library(reshape2)

library(ggplot2)

library(ggpubr)

expFile="input.txt"

riskFile="risk.txt"

geneFile="gene.txt"

rt=read.table(expFile, header=T, sep="\t", check.names=F)

rt=as.matrix(rt)

rownames(rt)=rt[,1]

exp=rt[,2:ncol(rt)]

dimnames=list(rownames(exp),colnames(exp))

data=matrix(as.numeric(as.matrix(exp)),nrow=nrow(exp),dimnames=dimnames)

data=avereps(data)

gene=read.table(geneFile, header=F, sep="\t", check.names=F)

sameGene=intersect(row.names(data),as.vector(gene[,1]))

data=t(data[sameGene,])

data=log2(data+1)

group=sapply(strsplit(row.names(data),"\\-"),"[",4)

group=sapply(strsplit(group,""),"[",1)

group=gsub("2","1",group)

data=data[group==0,]

row.names(data)=gsub("(.*?)\\-(.*?)\\-(.*?)\\-(.*?)\\-.*","\\1\\-\\2\\-\\3",row.names(data))

data=avereps(data)

risk=read.table(riskFile, sep="\t", header=T, check.names=F, row.names=1)

sameSample=intersect(row.names(data),row.names(risk))

rt1=cbind(data[sameSample,],risk[sameSample,])

rt1=rt1[,c(sameGene,"risk")]

sigGene=c()

for(i in colnames(rt1)[1:(ncol(rt1)-1)]){

if(sd(rt1[,i])<0.001){next}

wilcoxTest=wilcox.test(rt1[,i] ~ rt1[,"risk"])

pvalue=wilcoxTest$p.value

if(wilcoxTest$p.value<0.05){

sigGene=c(sigGene, i)

}

}

sigGene=c(sigGene, "risk")

rt1=rt1[,sigGene]

rt1=melt(rt1,id.vars=c("risk"))

colnames(rt1)=c("risk","Gene","Expression")

group=levels(factor(rt1$risk))

rt1$risk=factor(rt1$risk, levels=c("Low","High"))

comp=combn(group,2)

my_comparisons=list()

for(j in 1:ncol(comp)){my_comparisons[[j]]<-comp[,j]}

boxplot=ggboxplot(rt1, x="Gene", y="Expression", fill="risk",

xlab="",

ylab="Expression",

legend.title="Risk",

width=0.8,

palette = c("#0066FF", "#FF0000") )+

rotate_x_text(50)+

stat_compare_means(aes(group=risk),

method="wilcox.test",

symnum.args=list(cutpoints=c(0, 0.001, 0.01, 0.05, 1), symbols=c("***", "**", "*", "ns")), label="p.signif")

pdf(file="checkpoint.diff.pdf", width=6, height=4)

print(boxplot)

dev.off()

#multiGSEA

library(plyr)

library(ggplot2)

library(grid)

library(gridExtra)

files=grep(".tsv",dir(),value=T)

data = lapply(files,read.delim)

names(data) = files

dataSet = ldply(data, data.frame)

dataSet$pathway = gsub(".tsv","",dataSet$.id)

gseaCol=c("#58CDD9","#7A142C","#5D90BA","#431A3D","#91612D","#6E568C","#E0367A","#D8D155","#64495D","#7CC767","#223D6C","#D20A13","#FFD121","#088247","#11AA4D","#FFB6C1","#B0C4DE","#483D8B","#6A5ACD","#87CEEB")

pGsea=ggplot(dataSet,aes(x=RANK.IN.GENE.LIST,y=RUNNING.ES,colour=pathway,group=pathway))+

geom_line(size = 1.5) + scale_color_manual(values = gseaCol[1:nrow(dataSet)]) +

labs(x = "", y = "Enrichment Score", title = "") + scale_x_continuous(expand = c(0, 0)) +

scale_y_continuous(expand = c(0, 0),limits = c(min(dataSet$RUNNING.ES - 0.02), max(dataSet$RUNNING.ES + 0.02))) +

theme_bw() + theme(panel.grid = element_blank()) + theme(panel.border = element_blank()) + theme(axis.line = element_line(colour = "black")) + theme(axis.line.x = element_blank(),axis.ticks.x = element_blank(),axis.text.x = element_blank()) +

geom_hline(yintercept = 0) +

guides(colour = guide_legend(title = NULL)) + theme(legend.background = element_blank()) + theme(legend.key = element_blank())+theme(legend.key.size=unit(0.5,'cm'))

pGene=ggplot(dataSet,aes(RANK.IN.GENE.LIST,pathway,colour=pathway))+geom_tile()+

scale_color_manual(values = gseaCol[1:nrow(dataSet)]) +

labs(x = "Hight Risk<----------->Low Risk", y = "", title = "") +

scale_x_discrete(expand = c(0, 0)) + scale_y_discrete(expand = c(0, 0)) +

theme_bw() + theme(panel.grid = element_blank()) + theme(panel.border = element_blank()) + theme(axis.line = element_line(colour = "black"))+

theme(axis.line.y = element_blank(),axis.ticks.y = element_blank(),axis.text.y = element_blank())+ guides(color=FALSE)

gGsea = ggplot_gtable(ggplot_build(pGsea))

gGene = ggplot_gtable(ggplot_build(pGene))

maxWidth = grid::unit.pmax(gGsea$widths, gGene$widths)

gGsea$widths = as.list(maxWidth)

gGene$widths = as.list(maxWidth)

dev.off()

pdf('multipleGSEA.pdf',

width=11,

height=6)

par(mar=c(5,5,2,5))

grid.arrange(arrangeGrob(gGsea,gGene,nrow=2,heights=c(.8,.3)))

dev.off()

#Heatmap

library(pheatmap)

expFile="input.txt"

geneFile="gene.txt"

rt=read.table(expFile,header=T,sep="\t",row.names=1,check.names=F)

geneRT=read.table(geneFile,header=F,sep="\t",check.names=F)

hmExp=rt[as.vector(geneRT[,1]),]

hmExp=log2(hmExp+0.1)

Type=c(rep("N",conNum),rep("T",treatNum))

names(Type)=colnames(hmExp)

Type=as.data.frame(Type)

pdf(file="heatmap.pdf",height=4.2,width=14)

pheatmap(hmExp,

annotation=Type,

color = colorRampPalette(c("blue", "white", "red"))(50),

cluster_cols =F,

show_colnames = F,

show_rownames = T,

scale="row",

fontsize = 12,

fontsize_row=10,

fontsize_col=10)

dev.off()

#Forest map

library(survival)

coxFile="input.txt"

geneFile="genes.txt"

rt=read.table(coxFile,header=T,sep="\t",row.names=1,check.names=F)

geneRT=read.table(geneFile,header=F,sep="\t",check.names=F)

rt=rt[as.vector(geneRT[,1]),]

gene=rownames(rt)

hr=sprintf("%.3f",rt$"HR")

hrLow=sprintf("%.3f",rt$"HR.95L")

hrHigh=sprintf("%.3f",rt$"HR.95H")

Hazard.ratio=paste0(hr,"(",hrLow,"-",hrHigh,")")

pVal=ifelse(rt$pvalue<0.001, "<0.001", sprintf("%.3f", rt$pvalue))

pdf(file="forest.pdf", width =8,height = 6)

n=nrow(rt)

nRow=n+1

ylim=c(1,nRow)

layout(matrix(c(1,2),nc=2),width=c(3,2))

xlim = c(0,3)

par(mar=c(4,2.5,2,1))

plot(1,xlim=xlim,ylim=ylim,type="n",axes=F,xlab="",ylab="")

text.cex=0.8

text(0,n:1,gene,adj=0,cex=text.cex)

text(1.5-0.5*0.2,n:1,pVal,adj=1,cex=text.cex);text(1.5-0.5*0.2,n+1,'pvalue',cex=text.cex,font=2,adj=1)

text(3,n:1,Hazard.ratio,adj=1,cex=text.cex);text(3,n+1,'Hazard ratio',cex=text.cex,font=2,adj=1,)

par(mar=c(4,1,2,1),mgp=c(2,0.5,0))

xlim = c(0,max(as.numeric(hrLow),as.numeric(hrHigh)))

plot(1,xlim=xlim,ylim=ylim,type="n",axes=F,ylab="",xaxs="i",xlab="Hazard ratio")

arrows(as.numeric(hrLow),n:1,as.numeric(hrHigh),n:1,angle=90,code=3,length=0.05,col="black",lwd=1)

abline(v=1,col="black",lty=2,lwd=1)

boxcolor = ifelse(as.numeric(hr) > 1, 'red', 'blue')

points(as.numeric(hr), n:1, pch = 15, col = boxcolor, cex=1.3)

axis(1)

dev.off()

#Circos map

options(stringsAsFactors=F)

library(corrplot)

library(circlize)

inputFile="input.txt"

outFile="circos.pdf"

rt=read.table(inputFile,sep="\t",header=T,check.names=F,row.names=1)

rt=t(rt)

cor1=cor(rt)

col = c(rgb(1,0,0,seq(1,0,length=32)),rgb(0,1,0,seq(0,1,length=32)))

cor1[cor1==1]=0

c1 = ifelse(c(cor1)>=0,rgb(1,0,0,abs(cor1)),rgb(0,1,0,abs(cor1)))

col1 = matrix(c1,nc=ncol(rt))

pdf(outFile,width=7,height=7)

par(mar=c(2,2,2,4))

circos.par(gap.degree=c(3,rep(2, nrow(cor1)-1)), start.degree = 180)

chordDiagram(cor1, grid.col=rainbow(ncol(rt)), col=col1, transparency = 0.5, symmetric = T)

par(xpd=T)

colorlegend(col, vertical = T,labels=c(1,0,-1),xlim=c(1.1,1.3),ylim=c(-0.4,0.4))

dev.off()

circos.clear()

#Immune type

library(limma)

library(ggplot2)

library(reshape2)

expFile="input.txt"

subtypeFile="Subtype_Immune_Model_Based.txt"

exp=read.table(expFile, header=T,sep="\t",row.names=1,check.names=F)

exp=exp[(exp[,"Type"]=="Tumor"),]

exp=as.matrix(exp[,1:(ncol(exp)-2)])

row.names(exp)=gsub(".$","",row.names(exp))

exp=avereps(exp)

subtype=read.table(subtypeFile, header=T,sep="\t",row.names=1,check.names=F)

sameSample=intersect(row.names(subtype),row.names(exp))

subtype=subtype[sameSample,]

subtype=gsub(".+Immune |\\)","",subtype)

exp=exp[sameSample,]

exp=cbind(as.data.frame(exp),subtype)

outTab=data.frame()

geneSig=c()

for(gene in colnames(exp)[1:(ncol(exp)-1)]){

rt1=exp[,c(gene,"subtype")]

colnames(rt1)=c("expression","subtype")

ks=kruskal.test(expression ~ subtype, data = rt1)

p=ks$p.value

outTab=rbind(outTab,cbind(gene,pvalue=p))

Sig=ifelse(p<0.001,"***",ifelse(p<0.01,"**",ifelse(p<0.05,"*","")))

geneSig=c(geneSig,Sig)

}

geneSig=c(geneSig,"")

colnames(exp)=paste0(colnames(exp),geneSig)

write.table(outTab,file="immuneType.xls",sep="\t",row.names=F,quote=F)

data=melt(exp)

colnames(data)=c("Subtype","Gene","Expression")

p1=ggplot(data,aes(x=Subtype,

y=Expression,

fill=Subtype))+

guides(fill=guide_legend(title="Immune Subtype"))+

labs(x = "Immune Subtype", y = "Gene expression")+

geom_boxplot()+ facet_wrap(~Gene,nrow =1)+ theme_bw()

pdf(file="immuneType.pdf",width=8,height=5)

print(p1)

dev.off()

#The correlation between gene expression and drug sensitivity

drugFile="drug.txt"

expFile="expression.txt"

gene="gene1"

expData=read.table(expFile,sep="\t",header=T,check.names=F,row.names=1)

drugData=read.table(drugFile,sep="\t",header=T,check.names=F,row.names=1)

outputFile=paste(gene,".cor.xls",sep="")

outTab=data.frame()

for(drug in rownames(drugData)){

corTab=rbind(expData[gene,],drugData[drug,])

corTab=corTab[,apply(corTab,2,min)>-100]

x=as.numeric(corTab[gene,])

y=as.numeric(corTab[drug,])

Cor=cor(x,y)

Cor=round(Cor,3)

outTab=rbind(outTab,cbind(gene,drug,Cor))

}

corVal=as.numeric(as.vector(outTab[,3]))

corMean=mean(corVal)

corSd=sd(corVal)

zVal=(corVal-corMean)/corSd

pValue=2*pnorm(-abs(zVal))

wt=cbind(outTab,corMean,corSd,zVal,pValue)

wt=wt[order(wt$pValue),]

write.table(file=outputFile,wt,sep="\t",quote=F,row.names=F)
