# Supplementary figures and images for: Comprehensive Analysis Identified Mutation-Gene Signature Impacts the Prognosis Through Immune Function in Hepatocellular Carcinoma
Source: Front Oncol. 2022 Mar 4;12:748557. doi: 10.3389/fonc.2022.748557 (PMC8931204; doi:10.3389/fonc.2022.748557)

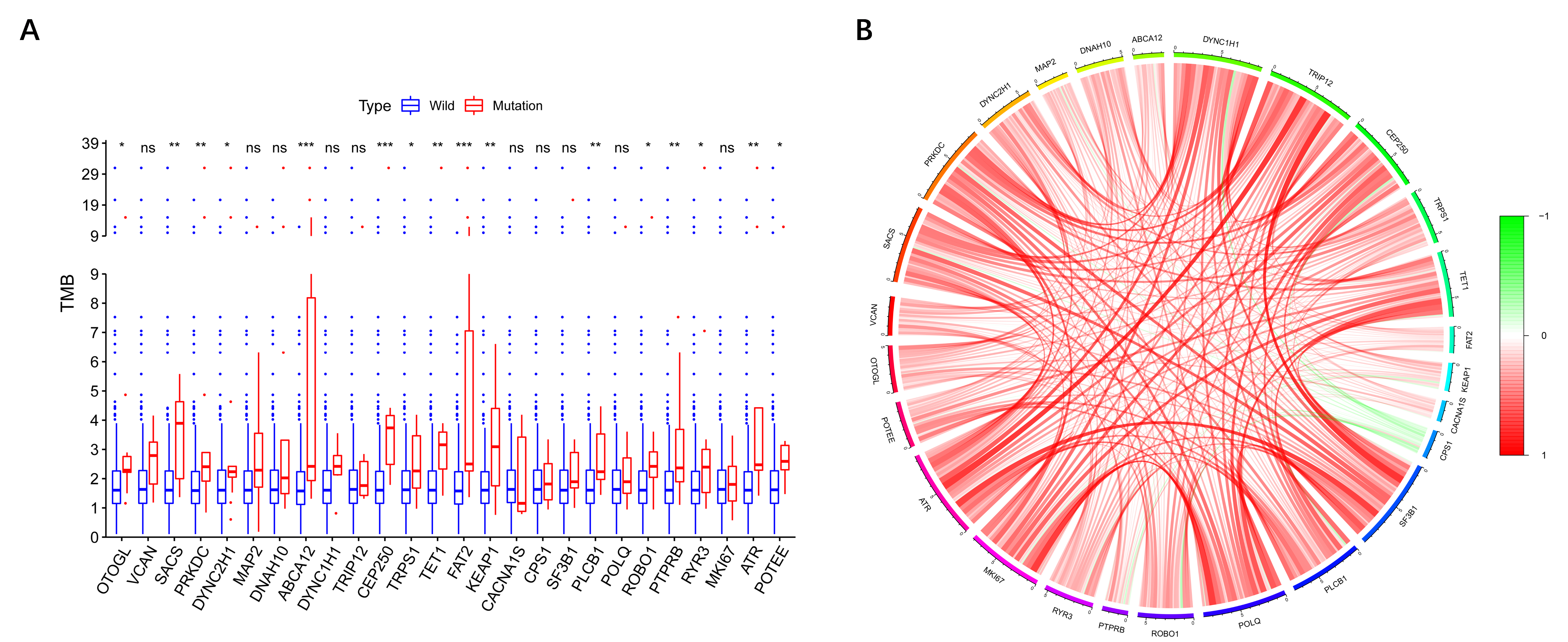

Supplement: Supplementary Figure 1 — The TMB difference between the patients with wild gene and patients with mutation gene and the correlation between the candidate genes. (A) Patients are grouped according to the gene wild type and mutation type, and then the TMB difference between each wild type group and mutation group was calculated. (B) The correlation between the 26 candidate genes. P values were showed as: ns, not significant; *P< 0.05; **P< 0.01; ***P< 0.001. [file Image_1.tif]

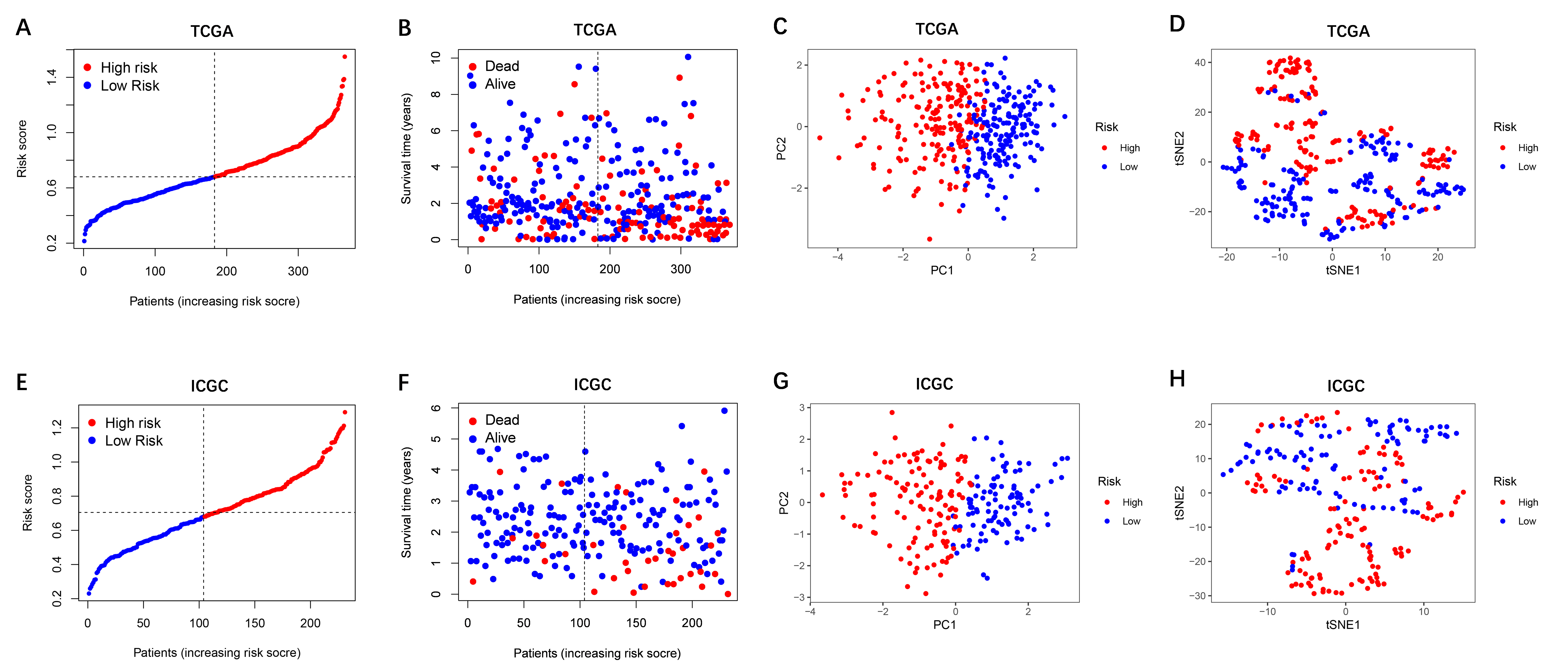

Supplement: Supplementary Figure 2 — Survival status of High- and low-risk patients. TCGA cohort (A-D), ICGC cohort (E-H). (A, B, E, F) Risk score distribution, patients’ survival status and time for high‐ and low‐risk groups in the entire set. (C, D, G, H) PCA and t-SNE analyses showed distinct clustering in high-risk patients and low-risk patients. [file Image_2.tif]

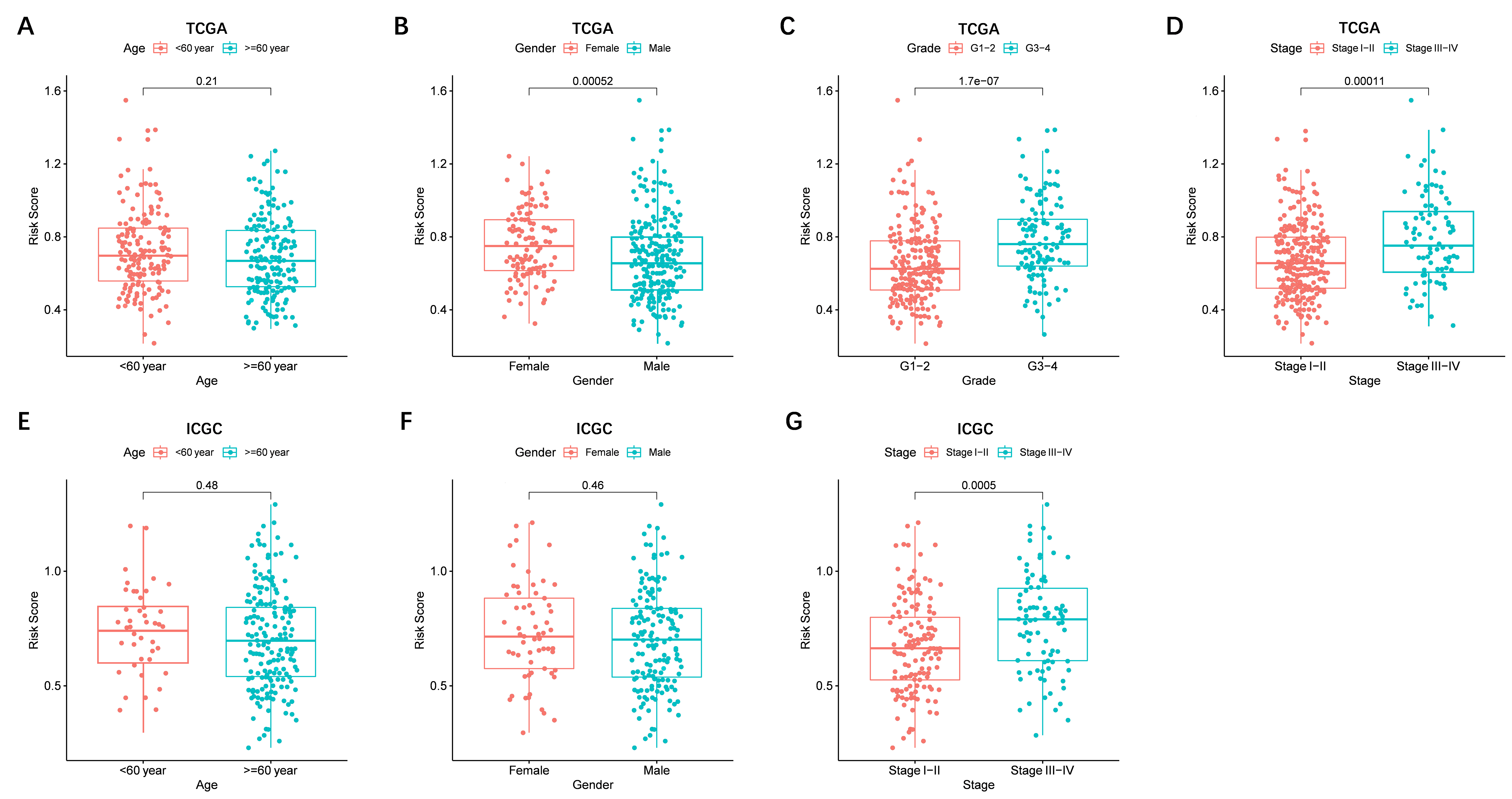

Supplement: Supplementary Figure 3 — The risk score in different groups stratified by clinical characteristics. [file Image_3.tif]

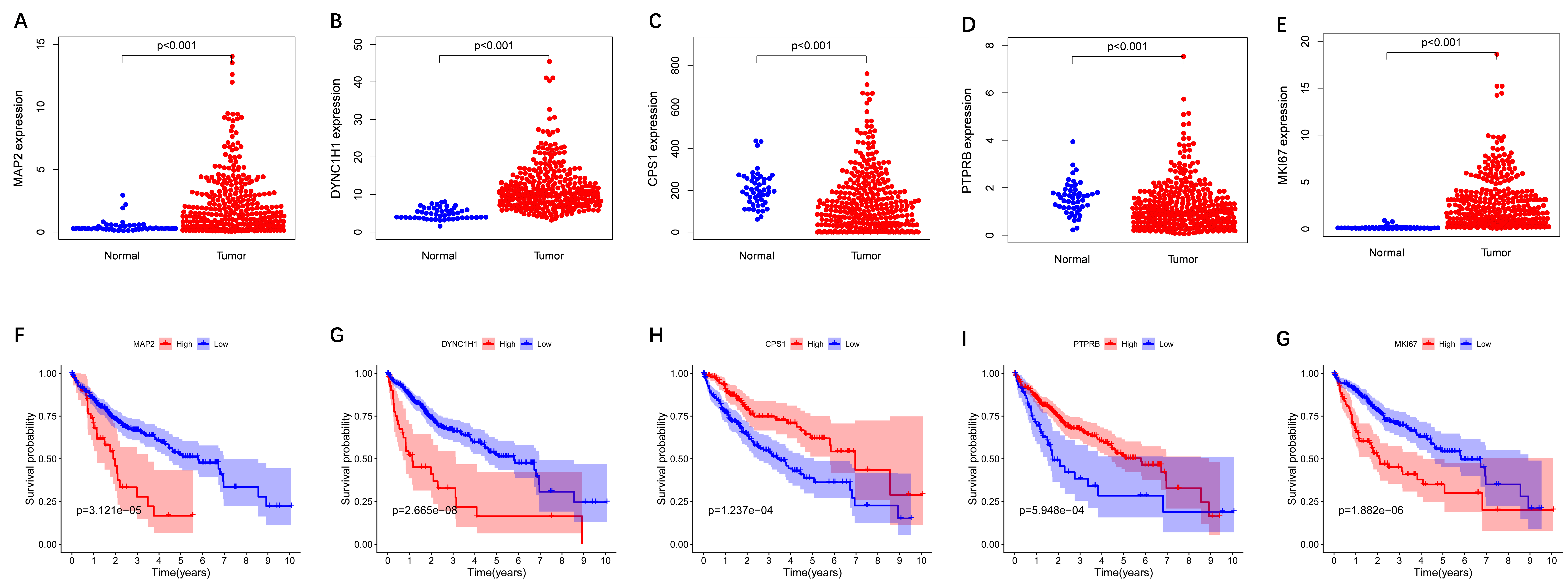

Supplement: Supplementary Figure 4 — The prognostic gene expression between HCC and para-cancerous tissues in the TCGA cohort and Kaplan–Meier survival curve of each prognostic gene for the patients with HCC in TCGA dataset. [file Image_4.tif]

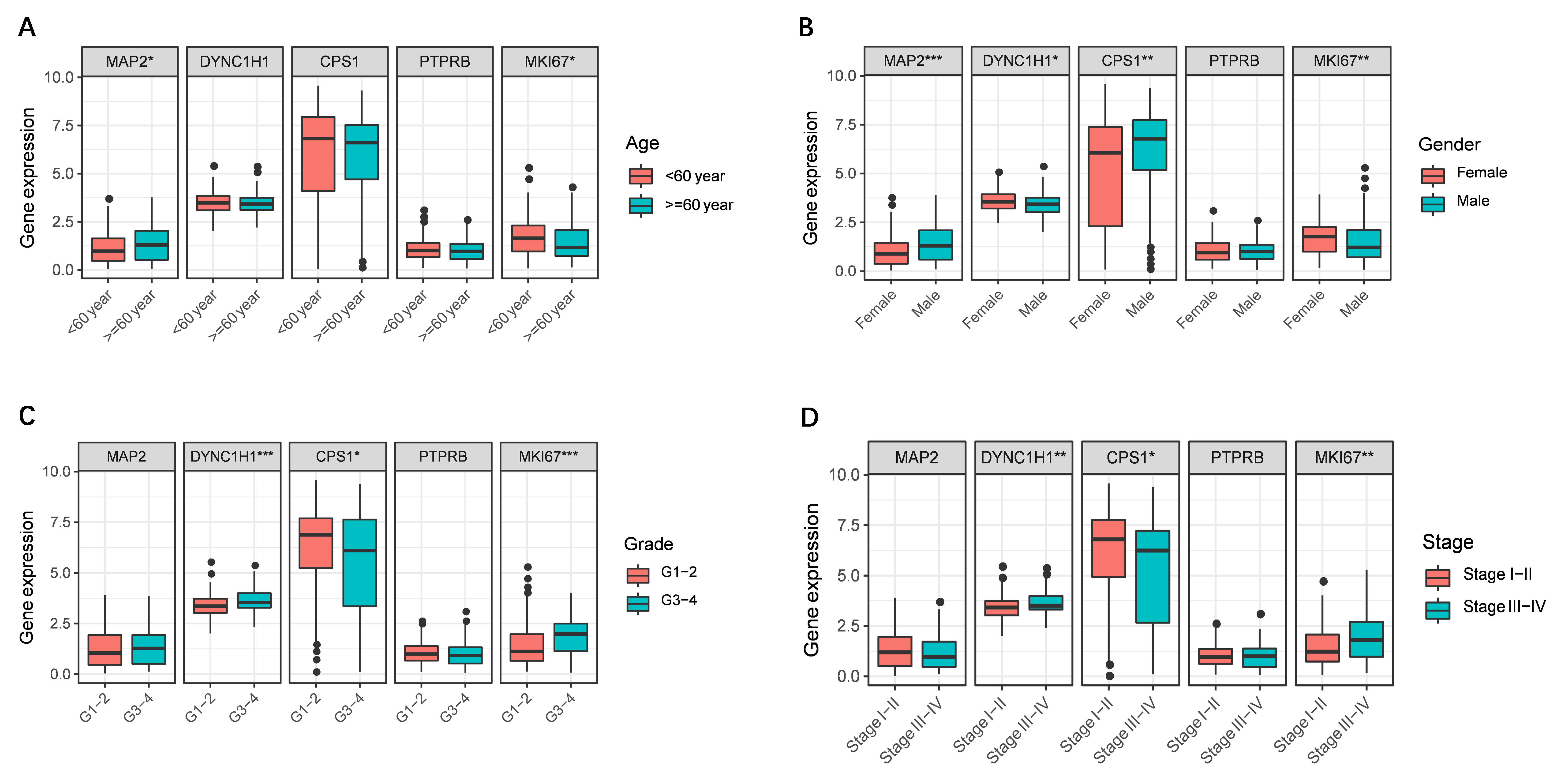

Supplement: Supplementary Figure 5 — The prognostic gene expression in different groups stratified by clinical characteristics. [file Image_5.tif]

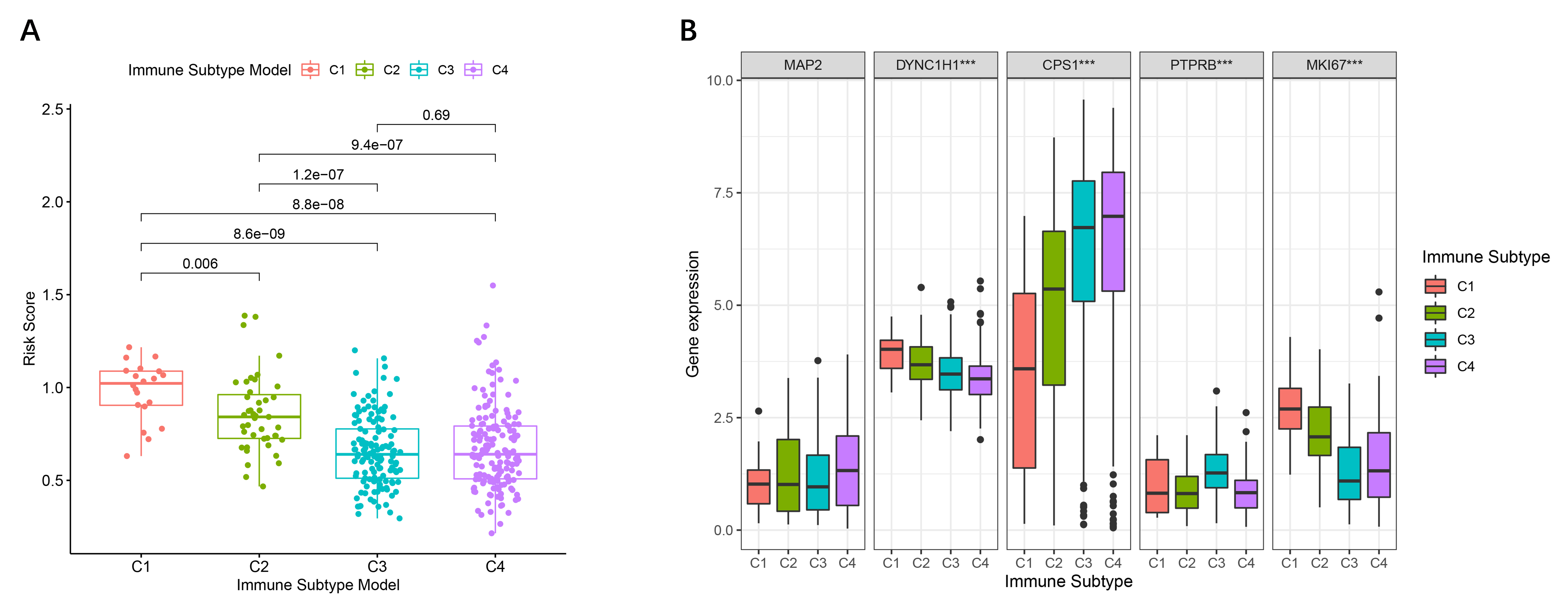

Supplement: Supplementary Figure 6 — The risk score and prognostic gene expression in different immune infiltration subtypes. [file Image_6.tif]

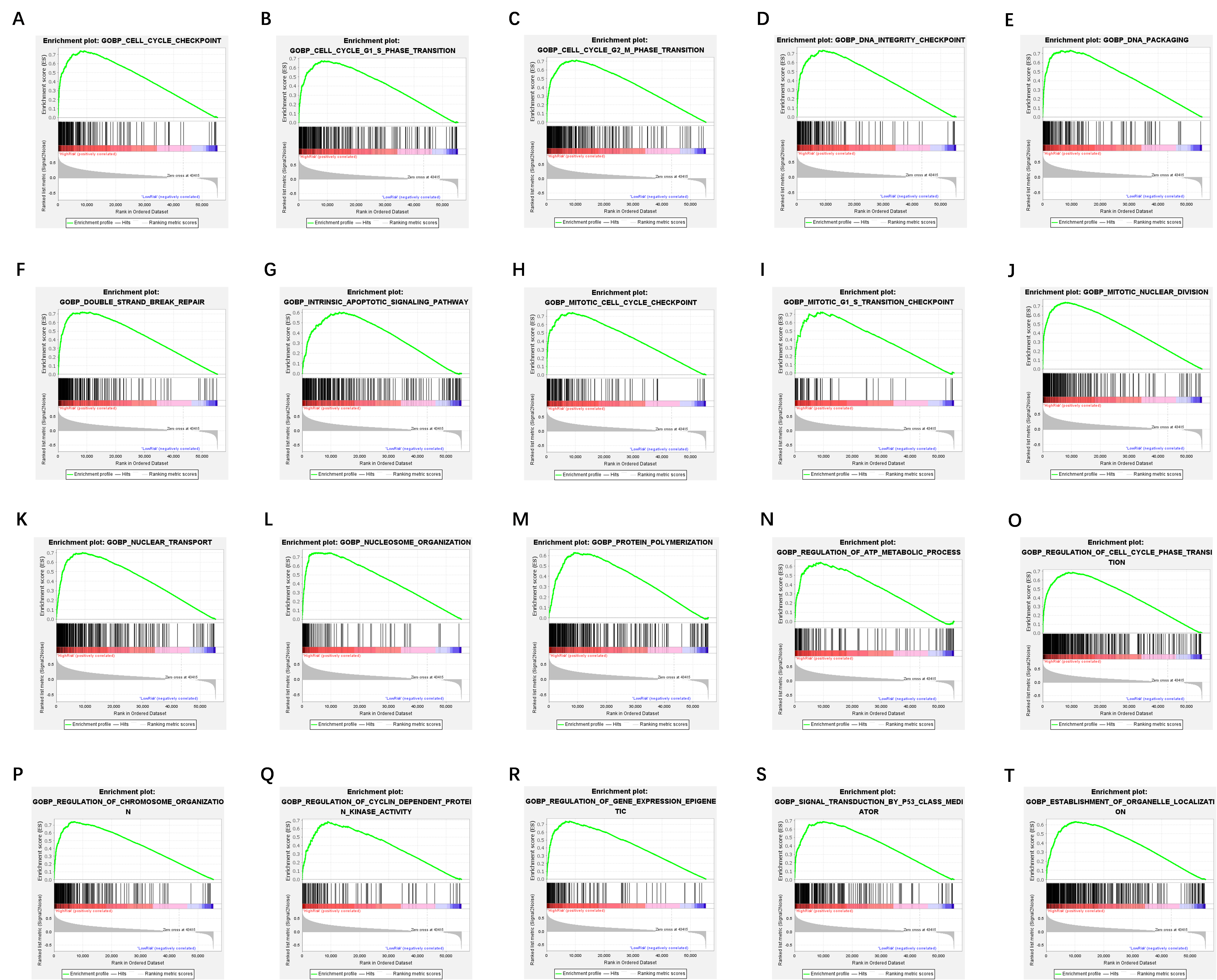

Supplement: Supplementary Figure 7 — The GSEA diagram of GO biological processes. [file Image_7.tif]

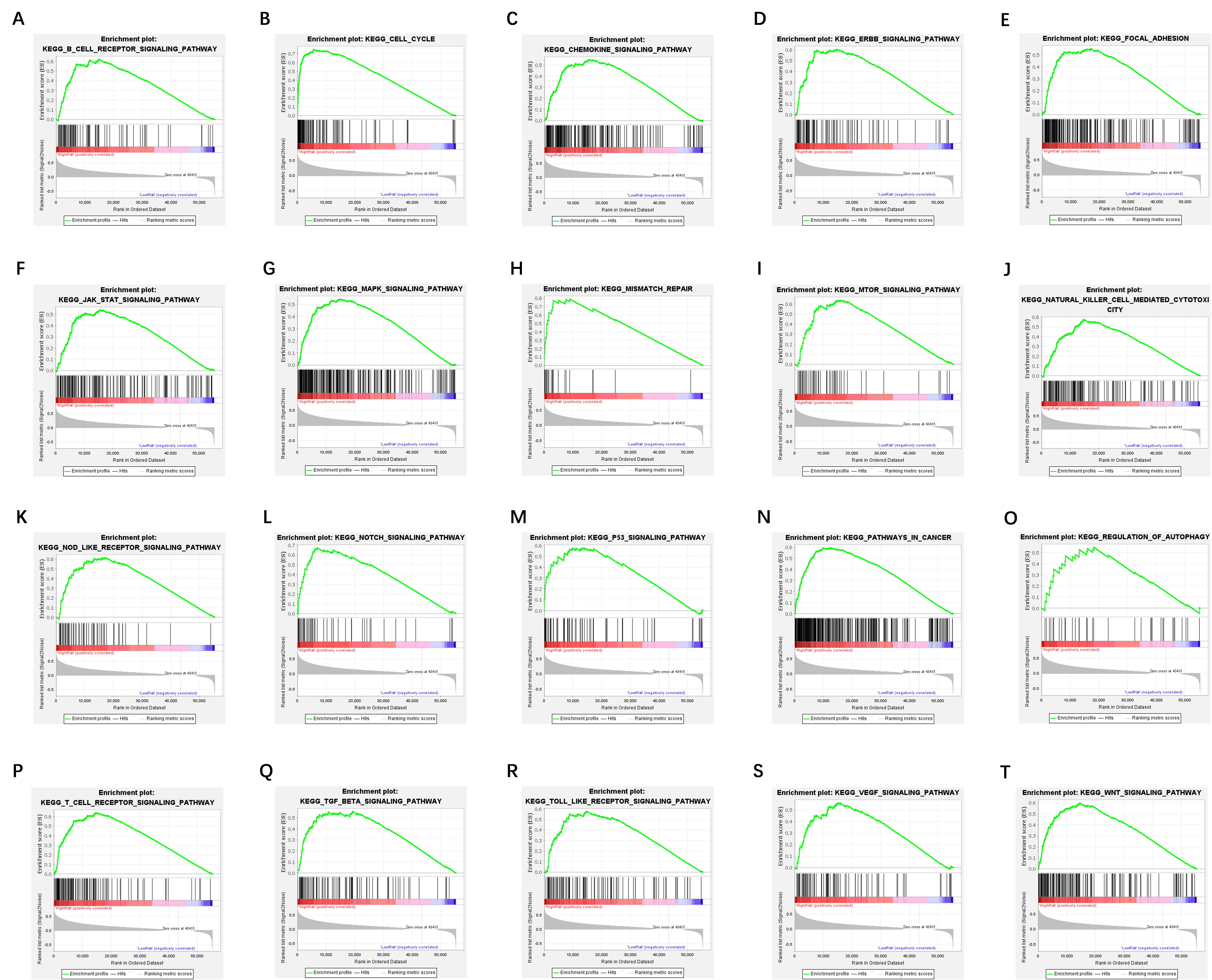

Supplement: Supplementary Figure 8 — The GSEA diagram of KEGG pathways. [file Image_8.tif]

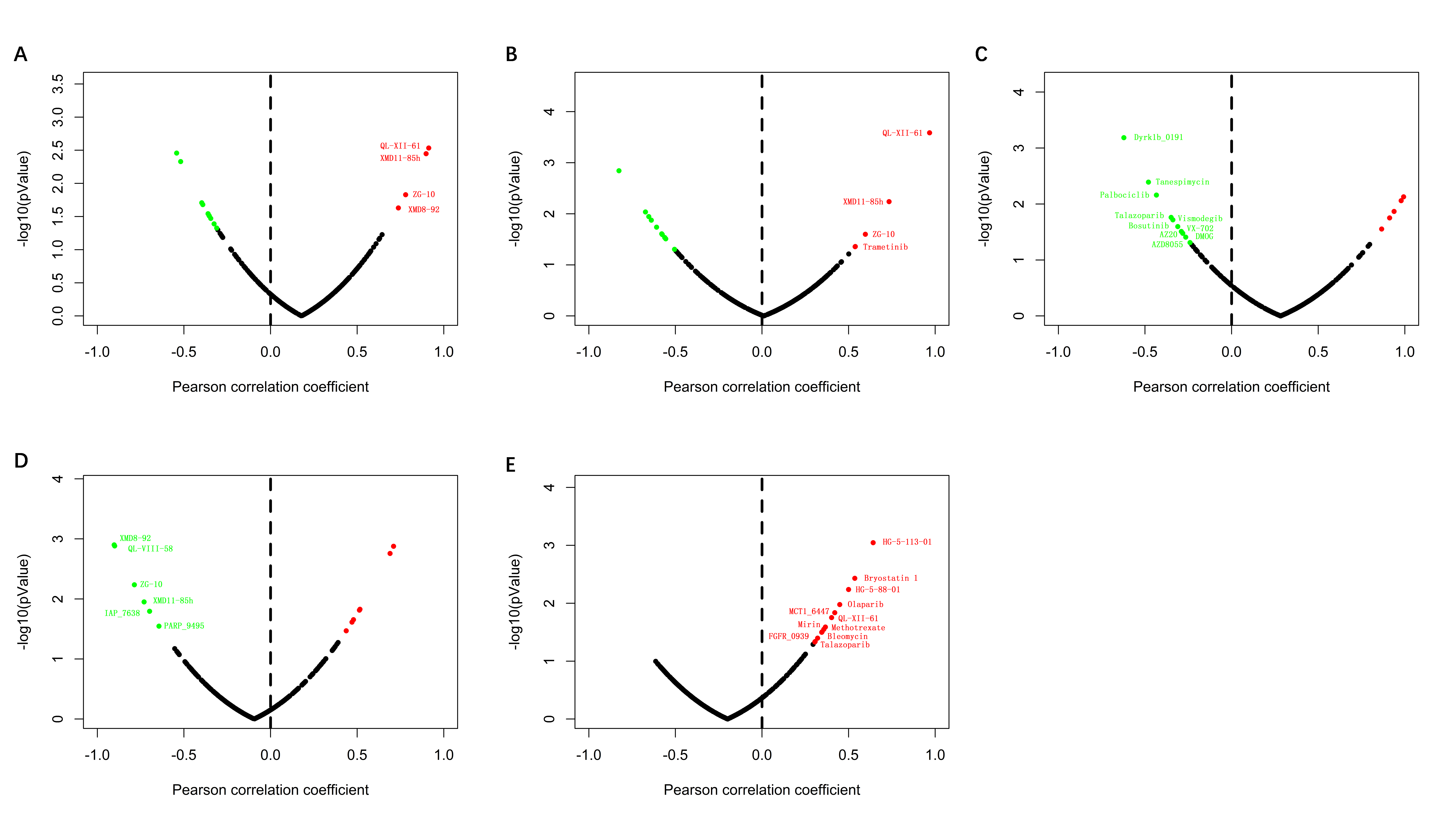

Supplement: Supplementary Figure 9 — The correlation of prognostic gene expression with drug resistance. MAP2. (B) DYNC1H1. (C) CPS1. (D) PTPRB. (E) MKI67. [file Image_9.tif]

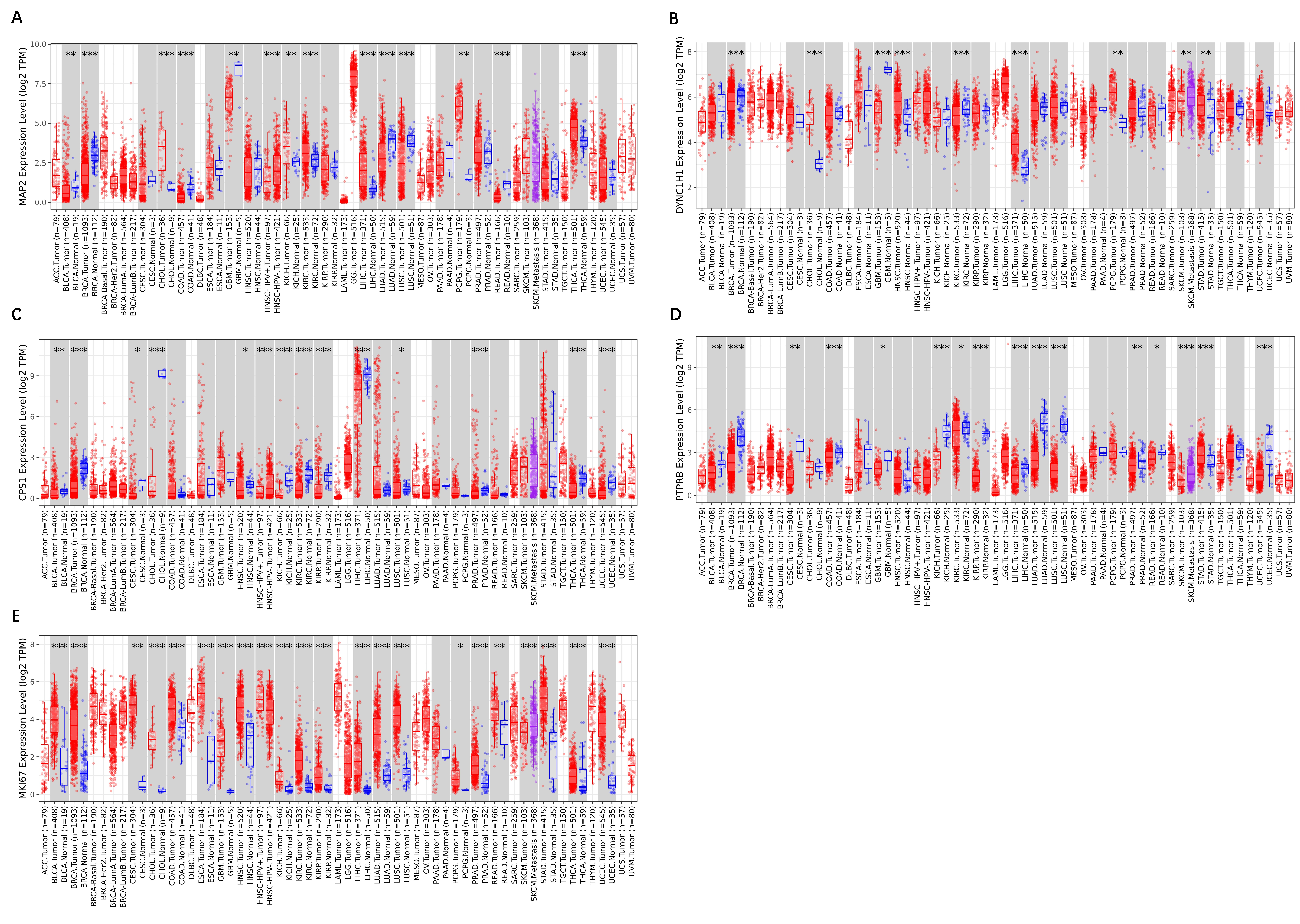

Supplement: Supplementary Figure 10 — Prognostic gene expression between pan-cancers and para-cancerous tissues. [file Image_10.tif]
